# Bacterial growth dynamics and pharmacokinetic–pharmacodynamic relationships of rifampicin and bedaquiline in BALB/c mice

**DOI:** 10.1111/bph.15688

**Published:** 2021-12-27

**Authors:** Morris Muliaditan, Oscar Della Pasqua

**Affiliations:** ^1^ Clinical Pharmacology & Therapeutics Group, School of Life and Medical Sciences University College London London UK; ^2^ Clinical Pharmacology, Modelling and Simulation GlaxoSmithKline Brentford UK

**Keywords:** bedaquiline, human dose selection, PKPD modelling, rifampicin, translational pharmacology, tuberculosis

## Abstract

**Background and Purpose:**

Translational efforts in the evaluation of novel anti‐tubercular drugs demand better integration of pharmacokinetic–pharmacodynamic data arising from preclinical protocols. However, parametric approaches that discriminate drug effect from the underlying bacterial growth dynamics have not been fully explored, making it difficult to translate and/or extrapolate preclinical findings to humans. This analysis aims to develop a drug‐disease model that allows distinction between drug‐ and system‐specific properties.

**Experimental Approach:**

Given their clinical relevance, rifampicin and bedaquiline were used as test compounds. A two‐state model was used to describe bacterial growth dynamics. The approach assumes the existence of fast‐ and slow‐growing bacterial populations. Drug effect on the growth dynamics of each subpopulation was characterised in terms of potency (EC_50_‐F and EC_50_‐S) and maximum killing rate.

**Key Results:**

The doubling time of the fast‐ and slow‐growing population was estimated to be 25 h and 42 days, respectively. Rifampicin was more potent against the fast‐growing (EC_50_‐F = 4.8 mg·L^−1^), as compared with the slow‐growing population (EC_50_‐S = 60.2 mg·L^−1^). Bedaquiline showed higher potency than rifampicin (EC_50_‐F = 0.19 mg·L^−1^; EC_50_‐S = 3.04 mg·L^−1^). External validation procedures revealed an effect of infection route on the apparent potency of rifampicin.

**Conclusion and Implications:**

Model parameter estimates suggest that nearly maximum killing rate is achieved against fast‐growing, but not against slow‐growing populations at the tested doses. Evidence of differences in drug potency for each subpopulation may facilitate the translation of preclinical findings and improve the dose rationale for anti‐tubercular drugs in humans.

**Abbreviations**
CFU: colony forming unit
HDA: high‐dose aerosol
LDA: low‐dose aerosol
MGT: mean generation time
PD: pharmacodynamic
PK: pharmacokinetic
TB: tuberculosis
VPC: visual predictive check

**What is already known**

- Pharmacokinetic–pharmacodynamic data integration is critical for the identification and ranking of anti‐tubercular drug candidates.
- Assessment of the antibacterial activity of drug combinations in infection models relies on empirical approaches.

**What does this study add**

- The growth dynamics model reveals the presence of fast‐ and slow‐growing subpopulations of *Mycobacterium tuberculosis*.
- The two subpopulations show different degrees of susceptibility to the antibacterial drug effects.

**What is the clinical significance**

- Evidence of differences in drug potency for bacterial subpopulations may improve dose selection in humans.
- This approach enables identification of suitable anti‐tubercular drug combinations prior to clinical testing in patients.

## 1 INTRODUCTION

A range of experimental models are available for the evaluation of the anti‐bacterial activity of anti‐tubercular drugs (Nuermberger, 2017). Irrespective of the differences between disease conditions in humans and an experimental model, effective integration of pharmacokinetic (PK) and pharmacodynamic (PD) data from well‐designed preclinical studies can provide valuable information for the selection of the optimal dose and companion drugs for combination therapy in patients (Franzblau et al., 2012; Gumbo et al., 2015). However, as drug exposure is often not assessed in most models, dose–response curves are derived without understanding of the underlying pharmacokinetic–pharmacodynamic (PKPD) relationships. Moreover, little attention is paid to the implications of phenotypical differences, bacterial burden, and growth dynamics, as compared with the disease in humans. For instance, *Mycobacterium tuberculosis* lipid‐rich phenotype can be up to 40 times more resistant to key antibiotics (Baron et al., 2018; Hammond et al., 2018). These differences could play a key role in the dose selection of novel regimens for the treatment of tuberculosis (TB) but are likely to be overlooked if one does not carefully consider the requirements for the collection and analysis of data from preclinical experimental protocols (Warner & Mizrahi, 2014).

Pharmacokinetic–pharmacodynamic (PKPD) modelling and simulation have evolved as an important tool for antibiotic development, providing quantitative estimates of antibiotic activity on bacterial killing rate (Nielsen et al., 2011). Recently, it has become evident that parameterisation of the factors that determine bacterial growth is also required to allow one to disentangle system‐specific properties from drug‐specific characteristics, thereby enabling the prediction of anti‐bacterial activity across different experimental conditions. Most importantly, the use of such a parametric approach allows knowledge to be reutilised and updated subsequently, as new data arise (Sheiner, 1997). To date, however, very little has been published regarding the use of modelling approaches that allow discrimination of drug effects from the growth dynamics of *M. tuberculosis* (Bartelink et al., 2017). This situation contrasts with ongoing efforts in the development of other antibiotics and antivirals, where PKPD modelling and simulation has been increasingly improving the quality and efficiency of preclinical research (Muliaditan et al., 2017).

Based on emerging evidence of the relevance of phenotypical differences *for M. tuberculosis*, it becomes evident that previous PKPD models, as the one proposed by Bartelink et al. (2017), may have limitations given that they do not provide a sufficiently physiological representation of the bacterial growth dynamics in humans and animals (Driver et al., 2012; Hoff et al., 2011; Sloan et al., 2015); that is, parameter estimates do not reflect the co‐existence of fast‐ and slow‐growing subpopulations or potential differences in sensitivity (Baron et al., 2018; de Steenwinkel et al., 2010; Mitchison, 1985). In fact, fitting of experimental data with such a model may be inadequate to differentiate the bactericidal and sterilising or time‐dependent activity of a drug. More recently, a three‐state model for PKPD analyses of preclinical experiment in TB has been proposed (Chen, Ortega, et al., 2017; Chen, Wicha, et al., 2017; Clewe et al., 2016). In this case, the approach accounts for three *M. tuberculosis* populations: fast, slow, and non‐replicating. While it attempts to incorporate growth dynamics during the course of infection, its implementation may be challenging, as evidence of the three different subpopulations cannot be obtained from total bacterial count, which is usually available experimentally. Hence, identifiability issues may arise during (re‐)estimation of the disease and PKPD model parameters. We argue, therefore, that a mechanism‐based parameterisation is still lacking, which allows discrimination of bacterial growth dynamics and other system‐specific properties from drug‐specific properties. In addition, such a model should be easily applied as a screening tool across different types of experimental protocols, enabling effective integration of preclinical PKPD data.

Here, we have used a meta‐analytical approach to develop a two‐state model in which bacterial growth dynamics and pharmacological activity are parameterised in a generic manner, as to allow its use to assess the anti‐bacterial effects of different compounds. Rifampicin and bedaquiline were used as test compounds to illustrate the application of the model as a tool for the characterisation, in a strictly quantitative manner, of differences in drug potency and efficacy. We anticipate that the availability of such a model will enhance the quality of candidate molecules selected during the screening for progression into development.

## 2 METHODS

### 2.1 Data source for the development of a bacterial growth dynamics model

Colony forming unit (CFU) counts per lung in untreated BALB/c mice infected with wild‐type H37Rv *M. tuberculosis* were extracted from publications by Zhang et al. (2012) and Swanson et al. (2016). More specifically, mean CFU count data from the control mouse group (marked as “WtRv” in the original publication; *N* = 3 per time point) infected with low‐dose *M. tuberculosis* (10^2^ CFU per lung) were extracted from Zhang et al., whereas regrowth data (as measured by change in CFU count) at the end of the 8‐week treatment with first‐line combination regimen were extracted from Swanson et al. (*N* = 4–5 mice per time point; individual CFU count from each mouse). Despite the availability of numerous publications, no additional literature data sets were deemed suitable for the purposes of the current investigation due to sparse sampling during the log phase of the infection (i.e., the period in which bacteria growth is exponential) and/or at stationary phase. These features were considered essential for a reliable estimation of the growth rate constants.

### 2.2 Data source for the parameterisation of drug effects

Pharmacokinetic data for rifampicin in BALB/c mice were extracted from a publication by Rosenthal et al. (2012). Time–kill curve data from infected mice with the H37Rv strain, as measured in lung CFU count drop following a 12‐week treatment with various doses of rifampicin (10–50 mg·kg^−1^), were extracted from Hu et al. (2015). PK data of bedaquiline were extracted from a publication by Tasneen et al. (2011). In their experiment, PK was characterised in BALB/c mice following single dose administration of 25 mg·kg^−1^ bedaquiline. In addition, time–kill curve data from infected mice with the H37Rv strain following a 6‐week treatment with 12.5 and 25 mg·kg^−1^ bedaquiline were extracted from Gupta et al. (2015). Intravenous (IV) inoculation and aerosol route were used in the rifampicin and bedaquiline experiments, respectively.

### 2.3 Bacterial growth dynamics model development

**FIGURE 1:**
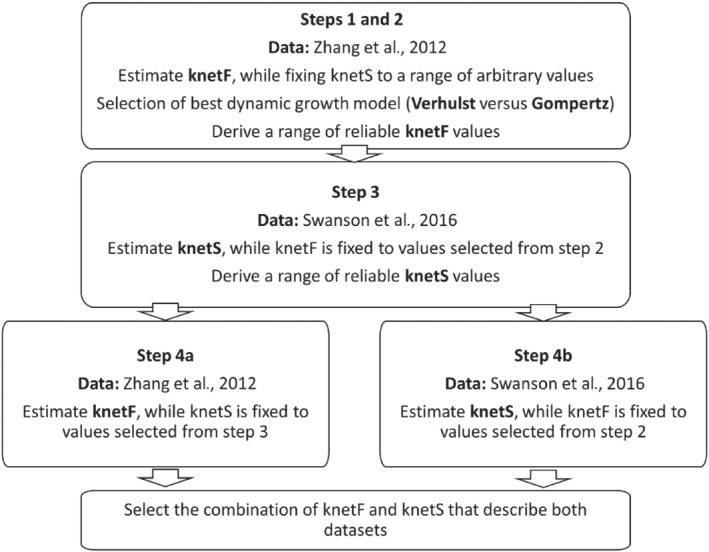
A schematic overview of the workflow used for the development of a bacterial growth dynamics model. The net growth rate constant of fast‐ and slow‐growing population (respectively knetF and knetS) was estimated from two publications (Zhang et al. and Swanson et al.) in a step‐wise calibration approach as outlined above. Colony forming unit (CFU) counts per lung over time in untreated infected BALB/c mice lung was measured for 8 weeks in the Zhang et al. experiment following aerosol infection with *Mycobacterium tuberculosis* (10^2^ CFU counts per lung). In the Swanson et al. experiment, CFU count per lung over time was measured in BALB/c mice for a period of 8 weeks post‐treatment with rifampicin, isoniazid, pyrazinamide, and ethambutol (given orally for 5 days per week for 2 months)

Here, we propose a model describing two bacterial populations that co‐exist at different ratios during the course of infection, namely, a fast‐growing (F) and a slow‐growing (S) *M. tuberculosis* subpopulation. We assumed that each bacterium can transition from fast to slow growing and vice versa. However, as CFU count data do not allow for simultaneous estimation of the growth rate constant of F and S subpopulations, a stepwise calibration approach was used for model building, in which one of the two growth rate constants was always fixed during the estimation step. A schematic overview of the workflow used for the calibration of the bacterial growth dynamics model is provided in Figure 1. In the experiment by Zhang et al., we assumed that all bacteria were fast‐growing at the start of infection. As such, this data set was used primarily to estimate the growth rate constant of the fast‐growing population. By contrast, in the Swanson experiment, we assumed that most fast‐growing bacteria were eradicated by the end of the 8‐week treatment with first‐line combination therapy. Consequently, all bacteria were considered to be slow growing at the start of the regrowth phase. This data set was used to estimate the growth rate constant of the slow‐growing population.

Initially, only the data set from Zhang et al. was used. Two dynamic growth models, the Verhulst model (Equation 1) and Gompertz model (Equation 2), were evaluated and formed the basis for the description of growth of the bacterial populations (Peleg & Corradini, 2011). The Gompertz survival function corresponds to an exponential mortality rate that increases with time. This contrasts with the Verhulst model, in which the initial stage of growth is approximately exponential, but then, as saturation begins, the growth slows down to a linear process, and at maturity growth stops completely:

(1) $$\text{Growth}=\left(1-\frac{\left(F+S\right)}{\text{BMAX}}\right),$$

(2) $$\text{Growth}=\log\left(\frac{\text{BMAX}}{F+S}\right),$$

where *F* or *S* is the total CFU count of each population and *BMAX* the carrying capacity of the system. Given that the growth and death rate of each population cannot be identified separately with the available experimental data, the net growth rate constant (knet) was estimated instead. This parameter corresponds to the difference between the growth rate constant *r* and death rate constant *d* (Equation 3). The mean generation time (MGT) in hours of each population can be subsequently calculated from knet according to Equation 4 (Garrett, 1978).

(3) knet=r−d,

(4) $$\mathrm{MGT}=\frac{\ln\left(2\right)}{\text{knet}}.$$

The growth function was subsequently incorporated into the differential equations below (Equations 5 and 6) to describe the growth dynamics of each population over time:

(5) $$\frac{\mathrm{dF}}{\mathrm{dt}}=\text{knetF}\cdot \text{Growth}\cdot F-\mathrm{kFS}\cdot F+\mathrm{kSF}\cdot S,$$

(6) $$\frac{\mathrm{dS}}{\mathrm{dt}}=\text{knetS}\cdot \text{Growth}\cdot S+\mathrm{kFS}\cdot F-\mathrm{kSF}\cdot S,$$

where *knetF* or *knetS* are the net growth rate constants of the F and S population, respectively, *Growth* the dynamic growth function as described in Equation 1 or 2, and *kFS* and *kSF* the rate constants describing the transfer from one growth state to another. Given that kFS and kSF cannot be identified from the available experimental data, both rates were fixed to the respective net growth rates constants (knetF and knetS), which in turn changed proportionally to the ratio between the total *M. tuberculosis* (equal to the sum of F and S population) and the system carrying capacity, BMAX (Equations 7 and 8).

(7) $$\mathrm{kFS}=\text{knetF}\cdot \left(\frac{F+S}{\text{BMAX}}\right),$$

(8) $$\mathrm{kSF}=\text{knetS}\cdot \left(\frac{F+S}{\text{BMAX}}\right).$$

The growth dynamics model that yielded the lowest minimum value of objective function (MVOF) was selected for the subsequent steps, during which the growth rate constants of fast‐ and slow‐growing *M. tuberculosis* population was estimated separately, as these parameters could not be identified simultaneously from the available experimental data sets. Further details of the analysis can be found in the Supporting Information.

### 2.4 Parameterisation of the effects of rifampicin and bedaquiline

**FIGURE 2:**
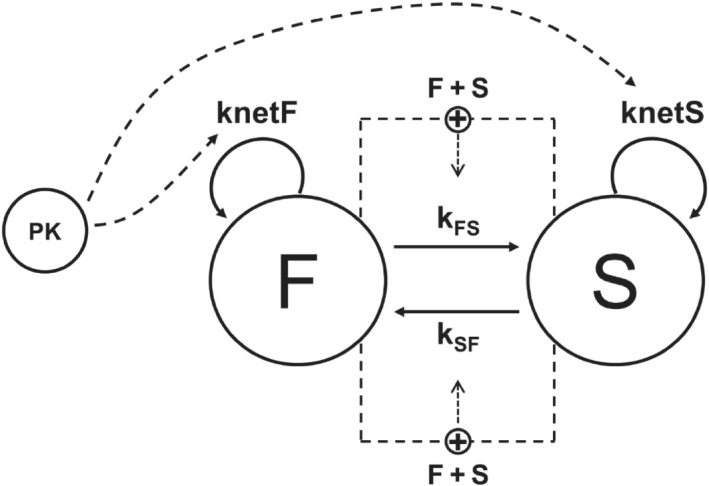
Schematic overview of the PKPD model of rifampicin and bedaquiline. Bacterial growth dynamics in the BALB/c mice, as measured by colony forming unit (CFU) counts, was described using a two‐state model which assumed the existence of fast‐growing (F) and slow‐growing (S) *Mycobacterium tuberculosis* populations with growth rate constants knetF and knetS, respectively. Each population can transfer from one growth state to another, as defined by transfer rate constants kFS and kSF. Both rate constants were fixed to knetF and knetS which changed proportionally to the ratio between F + S and the system carrying capacity (BMAX). Drugs were assumed to be active against both populations. PK represents the average steady‐state concentrations of rifampicin or bedaquiline per dosing interval

Given the known mechanism of action of rifampicin and bedaquiline, serum steady‐state concentrations were used as metrics of drug exposure at the target tissue (Alffenaar et al., 2019). As such, we have assumed that eventual interindividual differences in the concentration versus time profile would not have implications for the overall bactericidal activity and could therefore be considered as the most suitable parameter for the evaluation of drug effects on bacterial growth dynamics. Given the experimental limitations, we have also assumed that interindividual variability in PK in rodents was minor, and exposure was not significantly different between experimental protocols. Details of the pharmacokinetic analysis in mice can be found in the Supporting Information. Drug effect was parameterised as described below in Equations 9 and 10:

(9) $$\frac{\mathrm{dF}}{\mathrm{dt}}=\left(\text{knetF}\cdot \text{Growth}-\frac{\text{Emax}\cdot \mathrm{Css},\mathrm{av}}{{\mathrm{EC}}_{50}\left(F\right)+\mathrm{Css},\mathrm{av}}\right)\cdot F-\mathrm{kFS}\cdot F+\mathrm{kSF}\cdot S,$$

(10) $$\frac{\mathrm{dS}}{\mathrm{dt}}=\left(\text{knetF}\cdot \text{Growth}-\frac{\text{Emax}\cdot \mathrm{Css},\mathrm{av}}{{\mathrm{EC}}_{50}\left(S\right)+\mathrm{Css},\mathrm{av}}\right)\cdot S+\mathrm{kFS}\cdot F-\mathrm{kSF}\cdot S,$$

where *Emax* is the maximum killing rate of rifampicin or bedaquiline (assumed to be comparable or similar for both populations) and *EC_*50*_‐F* and *EC*_*50*_ *‐S* the potency against the fast‐ and slow‐growing *M. tuberculosis*, respectively. *Css, av* is the average steady‐state concentration derived from the predicted (simulated) area under the concentration versus time curve (AUC_0‐24_) divided by the dosing interval (24 h). A schematic overview of the final drug‐disease model structure is shown in Figure 2. Visual predictive checks (VPCs) were used as a diagnostic tool to assess the performance of the model. VPCs (1000 iterations) were generated using the estimated parameters, with variation in the predicted CFU profiles coming from the estimated residual error. Interindividual variability could not be estimated, as only mean data were available for the purposes of this analysis.

### 2.5 Estimation of the maximum killing rate

In contrast to retrospective data analysis, where model parameterisation is often descriptive, here, we attempted to develop a model that could be used prospectively as a tool for ranking and comparison of drug potency and bactericidal activity. Such a model is envisaged to improve the selection of suitable candidate molecules for progression into development. Taking into account the known correlation between the two drug‐specific parameters in the model, that is, *EC*_*50*_ *‐F*, *EC*_*50*_ *‐S*, and *Emax*, we have decided to establish a reference value for the maximum effect and focus on the estimation of apparent potencies for each compound. By fixing the maximum killing rate, potency estimates from different compounds should not be confounded by apparent, dose‐dependent Emax estimates.

In addition, based on evidence from additional experimental data (de Steenwinkel et al., 2013), it was assumed that the rifampicin doses administered in the experimental protocols which were used for model building would not allow for estimation of the true maximum bactericidal effect (Emax). In their publication, the authors have demonstrated that 160 mg·kg^−1^ was associated with zero CFU count within 3 weeks, while a much longer time to eradication was reported after administration of the 50 mg·kg^−1^ dose (Hu et al., 2015). This variation has prompted us to consider the use of a hypothetical time–kill curve as reference for the purposes of our analysis, ensuring a plausible, and yet accurate estimate of maximum killing, according to which zero CFU count can be obtained within 3 weeks of treatment (Figure S1). The maximum killing rate derived for rifampicin from this hypothetical data was fixed throughout the analysis.

It should be noted that the use of reference values does not prevent the estimation of Emax during the evaluation of the anti‐tubercular activity of another molecule.

### 2.6 External validation: Prediction of drug effect

To characterise the predictive performance of the model, additional experimental data in BALB/c mice were collected for rifampicin (Almeida et al., 2009; Hu et al., 2016; Rosenthal et al., 2012; Tasneen et al., 2008) and bedaquiline (Tasneen et al., 2015). Since the route of infection and onset of treatment are associated with significant differences between common murine experimental models in TB (Nuermberger, 2017), validation data sets included a variety of experimental protocols with different infection routes: high‐dose aerosol (HDA), low‐dose aerosol (LDA), and IV. These factors would have been included into an initial covariate model if a larger number of publications were available for the two compounds. The inclusion of different protocols represented therefore an attempt to assess the magnitude of such factors on the bacterial growth dynamics. Disease‐specific parameters (e.g., inoculum and BMAX) were fixed to the estimated values in each experiment. The remaining model parameters (e.g., EC_50_ and knet) were fixed to estimates from the initial analysis. Model performance was subsequently evaluated using VPCs, with variation in the predicted CFU profiles coming from estimated residual error from the training dataset.

### 2.7 Software

WebPlotDigitizer [RRID:SCR_013996] was used to extract concentration versus time profiles and CFU counts (in treated and untreated mice) from the publications (Rohatgi, 2017). Data analysis was performed using a non‐linear mixed effects approach as implemented in NONMEM 7.3 [RRID:SCR_016986] (ICON Development Solutions, Ellicott City, MD, USA) (Beal et al., 2009). Data manipulation, graphical, and statistical summaries were performed in R version 3.2.5 (R Core Team, 2016).

### 2.8 Nomenclature of targets and ligands

Key protein targets and ligands in this article are hyperlinked to corresponding entries in the common portal for data from the IUPHAR/BPS Guide to PHARMACOLOGY (http://www.guidetopharmacology.org) (Harding et al., 2018), and are permanently archived in the Concise Guide to PHARMACOLOGY 2021/22 (Alexander et al., 2019).

## 3 RESULTS

### 3.1 Bacterial growth dynamics model

As shown in Table S1 and Figure S2, the use of a Gompertz growth function performed significantly worse in terms of describing the available experimental data, as compared with the Verhulst model. Therefore, based on statistical criteria and on biological plausibility, the Verhulst model was selected as the most suitable parameterisation to describe the growth dynamics of the fast‐ and slow‐growing populations in the subsequent analyses. Details of the development steps for the model describing bacterial growth dynamics are summarised in the Supporting Information (Figures S3–S6, Table S2). Although the stationary phase of the experimental data by Zhang et al. was not fully described, the model was deemed adequate for the following reasons: (1) The log phase of the growth curve was adequately captured, and (2) the shape of the stationary phase in the data set from the experimental data by Zhang et al. seems to deviate from the patterns observed in other murine experiments (Figure S7).

**FIGURE 3:**
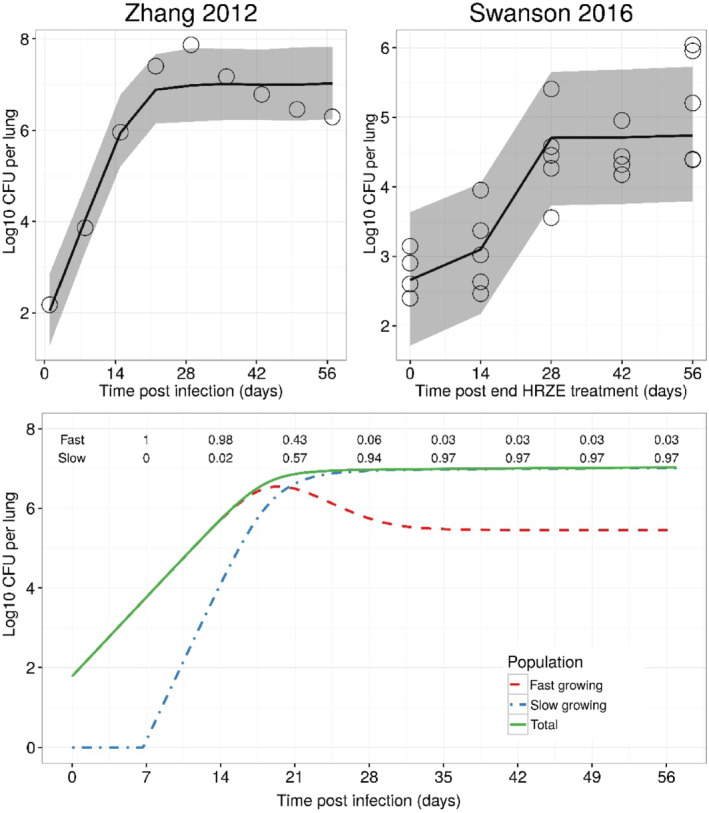
Visual predictive check of the final disease model (upper panel) and the model‐predicted theoretical dynamics of fast‐ and slow‐growing *Mycobacterium tuberculosis* over time (lower panel). Solid lines and shaded area represent the median and 90% prediction interval of the predicted log10 colony forming unit (CFU) per lung over time. Open circles represent the observed log10 CFU per lung in the publications by Zhang et al. (2012) and Swanson et al. (2016). The numbers above the lines correspond to the fraction of each population (i.e., fast‐ and slow‐growing) relative to the total bacterial load at 7, 14, 21, 28, 35, 42, 49, and 56 days after the onset of infection. HRZE, first‐line treatment consisting of rifampicin, isoniazid, pyrazinamide, and ethambutol

The predicted growth dynamics of each population over time is shown in Figure 3. These profiles correspond to an MGT of 25 h for the fast‐growing and 42 days for the slow‐growing population. In addition, these data indicate that the fast‐growing *M. tuberculosis* is the initial dominant population (e.g., 98% at 14 days post infection) in mice. Upon entering the stationary phase (i.e., after approximately 20 days post infection), most of the viable bacteria (97%) has transitioned into the slow‐growing state.

### 3.2 Effects of rifampicin and bedaquiline on growth dynamics

**FIGURE 4:**
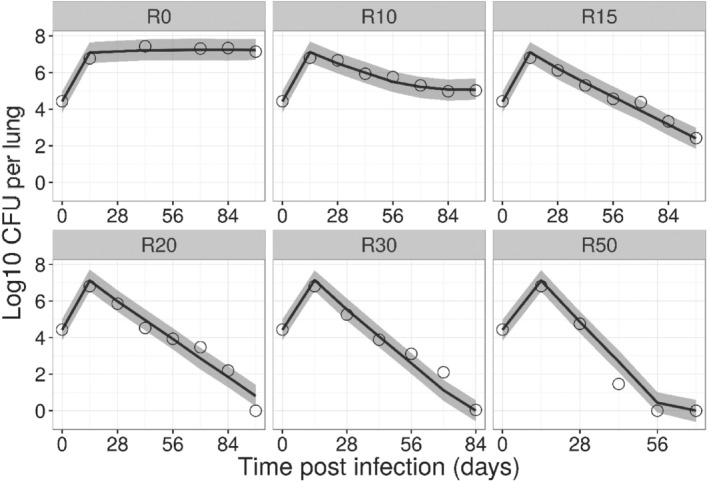
Visual predictive check of the PKPD model describing the effects of rifampicin (R) in BALB/c mice using experimental data from Hu et al. BALB/c mice were infected with H37Rv strain intravenously and were left untreated for 2 weeks to allow the infection to progress. Following this incubation period, treatment with doses of 0 (R0), 10 (R10), 15 (R15), 20 (R20), 30 (R30), or 50 (R50) mg·kg^−1^ rifampicin for 12 weeks (5 days per week) was initiated. Solid lines and shaded area represent the median and 90% prediction interval of the predicted log10 colony forming unit (CFU) per lung over time. Open circles represent the observed log10 CFU per lung

The VPCs describing the concentration versus time profiles for rifampicin and bedaquiline are shown in Figure S8. The maximum killing rate for rifampicin that corresponded to complete bacterial clearance within 3 weeks was estimated to be 0.0671 per hour (Figure S9). Estimated system‐specific parameters (e.g., inoculum and BMAX) in each experiment are shown in Table S3. In addition, our analysis showed that rifampicin is more potent against the F population (EC_50_‐F = 4.87 mg·L^−1^) as compared with the S population (EC_50_‐S = 60.2 mg·L^−1^). The VPCs showing model predictions for the rifampicin treatment groups are depicted in Figure 4.

**FIGURE 5:**
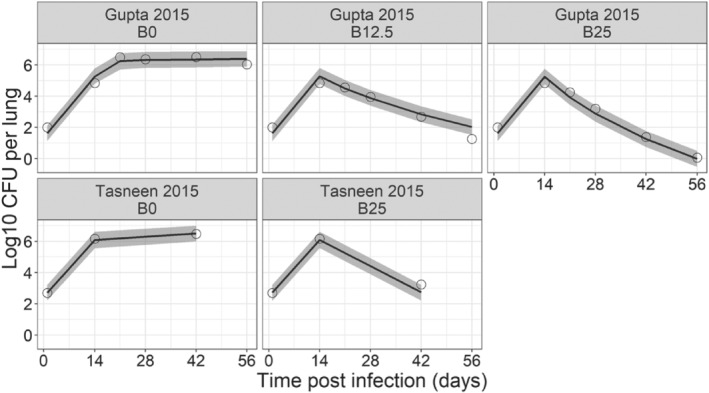
Visual predictive check of the PKPD model describing the effects of bedaquiline (B) in BALB/c mice using experimental data from Gupta et al. and Tasneen et al. BALB/c mice were infected with H37Rv strain via an aerosol route and were left untreated for 2 weeks. Following this incubation period, treatment with doses of 0 (B0), 12.5 (B12.5) and 25 (B25) mg.kg^−1^ bedaquiline was initiated and maintained for 4 (Tasneen et al.) or 6 (Gupta et al.) weeks (given 5 days per week). Solid lines and shaded area represent the median and 90% prediction interval of the predicted log10 colony forming unit (CFU) per lung over time. Open circles represent the observed log10 CFU per lung

For bedaquiline, EC_50_‐F and EC_50_‐S were estimated to be 0.202 and 2.74 mg·L^−1^ based on the experiment by Gupta et al. However, external validation of the integrated model describing the drug effect on bacterial growth dynamics showed slight overprediction of the anti‐bacterial activity of bedaquiline in the Tasneen experiment (data not shown). It was clear that the external validation data set for the bedaquiline model had limitations, and as such, we decided to re‐estimate the parameters with both data sets and use the integrated results as final estimates of EC_50_‐F and EC_50_‐S, which were 0.192 and 3.04 mg·L^−1^, respectively. Good predictive performance was observed for both bedaquiline treatment groups as shown in Figure 5. These findings furthermore reveal that bedaquiline is more potent against *M. tuberculosis* than rifampicin.

**FIGURE 6:**
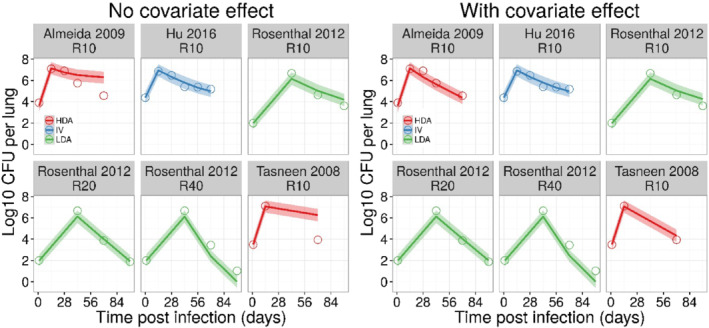
External validation of the in vivo rifampicin (R) PKPD model. As shown in the left panel, only BALB/c mice who were infected via the intravenous (IV) or low dose aerosol (LDA) route were predicted. Once a covariate effect of high‐dose aerosol (HDA) infection route was estimated, adequate predictions were achieved for all experimental data (right panel). Solid lines and shaded area represent the median and 90% prediction interval of the predicted log10 colony forming unit (CFU) per lung over time. Open circles represent the observed log10 CFU per lung. Numbers in the headers of each panel represent the rifampicin dose in mg·kg^−1^ (given 5 days per week)

From a methodological point of view, we were also confronted with the effect of different experimental procedures on growth dynamics. For instance, the initial results with the rifampicin parameters derived from the Hu experiment did not predict anti‐microbial activity in other experiments that used HDA infection route (Figure 6). Given the evidence of differences in microbial clearance after different routes and mode of infection (De Groote et al., 2011), infection route was included as a covariate factor on EC_50_‐F and EC_50_‐S (Figure 6). Apparent potency estimates in mice infected via the HDA route were higher than after IV infection. Estimated EC_50_ values were approximately 66% lower (i.e., EC_50_‐F and EC_50_‐S were 3.25 and 40.2 mg·L^−1^, respectively) as compared with experiments that used IV infection route. On the other hand, apparent potency estimates were comparable following LDA and IV infection route (i.e., EC_50_‐F and EC_50_‐S were 4.9 and 60.8 mg·L^−1^, respectively). An overview of the final model parameters for both drugs is presented in Table 1.

**TABLE 1:** Final parameter estimates of the growth dynamics model including drug‐specific parameter estimates for rifampicin and bedaquiline

Model parameters	Final estimate	
	Rifampicin	Bedaquiline
Pharmacokinetics^a^		
Absorption rate constant, Ka (h^−1^)	0.537	5.39^b^
Apparent central volume of distribution, Vc/F (ml)	8.22	312
Apparent peripheral volume of distribution, Vp/F (ml)	‐	298
Apparent clearance, CL/F (ml·h^−1^)	1.44	24.4
Apparent inter‐compartmental clearance, Q/F (ml·h^−1^)	‐	9.95
Bacterial growth dynamics^a^		
Growth rate constant ‐ fast‐growing population, knetF (h^−1^)	0.0272	0.0272
Growth rate constant ‐ slow‐growing population, knetS (h^−1^)	0.00068	0.00068
Carrying capacity in experiment, BMAX (log10 CFU per lung)	7.26	1.36
Inoculum in experiment (log10 CFU per lung)	4.41	6.41
Drug effects		
Maximum killing rate, Emax (h^−1^)	0.0671 (FIX)	0.0671 (FIX)
Potency against fast‐growing population (mg·L^−1^)		
IV infection route, EC_50_‐F_IV_	4.87	‐
HDA infection route, EC_50_‐F_HDA_ ^c^	3.25	0.192
LDA infection route, EC_50_‐F_LDA_ ^d^	4.97	‐
Potency against slow‐growing population (mg·L^−1^)		
IV infection route, EC_50_‐S_IV_	60.2	‐
HDA infection route, EC_50_‐S_HDA_ ^c^	40.2	3.04
LDA infection route, EC_50_‐S_LDA_ ^d^	61.4	‐
Additive residual error on CFU counts, σ (log10 CFU per lung)	0.36	0.31

Abbreviations: HDA, high‐dose aerosol; IV, intravenous; LDA, low‐dose aerosol.

^a^ Fixed during estimation of PKPD parameters.

^b^ Fixed to value as reported in Irwin et al. (2016).

^c^ Calculated from EC_50__HDA_ = EC_50__IV_ · Θ_HDA‐effect_, where Θ_HDA‐effect_ = 0.667.

^d^ Calculated from EC_50LDA_ = EC_50__IV_ · Θ_LDA‐effect_, where Θ_LDA‐effect_ = 1.02.

## 4 DISCUSSION

### 4.1 Parameterisation of bacterial growth dynamics

The assessment of bacterial growth dynamics in vivo represents a critical step towards the translation of drug effects from preclinical settings to humans. In contrast to previous PKPD modelling efforts, where data analysis is often aimed at the description of the anti‐tubercular activity of the compound in a parametric manner, the current model was parameterised bearing in mind its use as a screening tool for the selection of novel candidate molecules in a prospective manner. In fact, in the current investigation, we have shown how a model‐based meta‐analytical approach can be used to characterise *M. tuberculosis* growth dynamics across a range of experimental protocols and assess drug effects in a systematic manner, enabling a clear distinction between system (disease)‐ and drug‐specific properties. Moreover, differently from typical time‐kill curves, where empirical results are usually described by standard PKPD indices or by the change in CFU counts at the end of treatment, our approach allows for ranking and direct comparison of the potency of anti‐tubercular drugs.

It is also worth mentioning that disease‐specific model parameters have been identified, which make evident that two subpopulations of *M. tuberculosis* co‐exist, with different growth capacity. Although transition rates between subpopulations are not fully identifiable from typical CFU count data, we anticipate that these subpopulations may correspond to the lipid‐rich and lipid‐poor classification, as suggested by Hammond et al. (2015). Insights from a recent review of the concept of mycobacterial dormancy also support the presence of two distinct phenotypes, one of which is characterised by low metabolic activity, alteration of gene regulation with the accumulation of tri‐acylglycerides in intracellular lipid bodies, loss of acid fastness, and tolerance to antibiotics (Lipworth et al., 2016). This is further substantiated by evidence from live cell imaging experiments, which clearly show the presence of two subpopulations of *M. tuberculosis* (Vijay et al., 2019). Previous PKPD models have either disregarded heterogeneity (Bartelink et al., 2017) or defined different bacterial states, including non‐replicating subpopulations, but model parameters have not been correlated with an underlying microbiological substrate (Chen, Ortega, et al., 2017; Chen, Wicha, et al., 2017; Clewe et al., 2016). Interestingly, even though multistate model parameterisation was based on total CFU count, the authors do not discuss issues such as parameter identifiability, correlations, or sensitivity to different experimental conditions.

A closer look at the characteristics of the two subpopulations emerging from this analysis reveals that the predicted MGT of the fast‐growing population is approximately 25 h, which is in agreement with values reported previously (Bartelink et al., 2017). On the other hand, published MGT of the slow‐growing population was very different from the estimates obtained here (1019 h [42 days] compared with 69–158 h) (Aljayyoussi et al., 2017; Beste et al., 2009; Raffetseder et al., 2014). Apparently, this discrepancy may be explained by the fact that published data were derived from in vitro growth conditions where *M. tuberculosis* only exists as a single (slow‐growing) state, all bacteria grow in conditions that correspond to the extracellular space and growth is not affected by the presence of immune cells. By contrast, in mice, it has been shown that *M. tuberculosis* is present in more than one state (Driver et al., 2012; Hoff et al., 2011; Sloan et al., 2015). As such, in vitro parameter estimates seem to provide limited physiological representation of the in vivo growth dynamics.

Because of the choice of parameterisation, a generic model has been identified, which allows the description of drug effects across different protocols, including different drugs and dose levels. Of note is the fact that our model can account for the effect of inoculum route, initial bacterial load, and duration of infection on drug effect. In addition, despite the availability of limited data from experimental protocols currently used for drug screening, we have been able to identify drug‐specific parameters that affect the growth dynamics of both subpopulations (i.e., EC_50_ and Emax).

### 4.2 Drug effect across different experimental protocols

With regard to drug‐specific parameters, we have assumed fast equilibration between plasma and lung tissue and negligible interindividual variability in pharmacokinetics. Furthermore, it was assumed that, for standard of care drugs, fluctuation in peak and trough concentrations does not lead to significant differences in the overall bactericidal activity. Consequently, steady‐state concentrations varied over time only if metabolic inhibition or induction was known to occur and had been identified during the PK model development. However, it should be clear that, when available, individual pharmacokinetic data can be easily incorporated into the analysis and used instead of simulated population estimates.

In addition, it is important to consider the role of the immune system and innate response to infection when comparing potency estimates from different experimental protocols. As the proposed model parameterisation does not include immune system‐related factors as covariates, estimates of drug potency should be considered *apparent* and as such reflect the contribution of unmeasured factors. This is the most likely explanation why in our analysis, rifampicin was found to be more potent in the HDA model than in LDA and IV infected mice. Higher inoculum size has been associated with more immune cells in the lung (Myers et al., 2013) and consequently may have made the bacterial populations more susceptible to clearance in the HDA model (as compared to LDA infected mice). Similarly, rifampicin potency was different between IV and HDA models, despite similar inoculum size and bacterial load at the onset of treatment. We postulate that the lower potency in IV infected mice could be potentially attributed to presence of a considerable proportion of extra‐pulmonary *M. tuberculosis* (De Groote et al., 2011) and more severe lung tissue damage after onset of the infection (de Steenwinkel et al., 2011). These differences suggest varying contribution of immune cells in IV versus HDA infected mice.

Another key finding in this investigation refers to rifampicin potency estimates for each subpopulation, which appear to provide an answer to the ongoing debate regarding the need for higher doses in the clinic to achieve maximum bacterial clearance (van Ingen et al., 2011). Even though direct comparisons may not be possible, the potency estimates for bedaquiline reflect previous findings in vitro (Dhillon et al., 2010) and correlate with expected drug levels required for efficacy at clinical doses (van Heeswijk et al., 2014).

While only two compounds have been evaluated, the results obtained so far may open a new perspective for the characterisation of treatment effect and ranking of compounds during drug screening, in that selection criteria can be based on potency and maximum killing rates, as opposed to empirical comparisons using traditional PKPD indices or other metrics of anti‐bacterial activity. In addition, we envisage the possibility of applying this model for study protocol optimisation purposes. Given the model's ability to describe CFU count over time, it may be possible to shorten the duration of some experimental protocols, as parameter estimates may be sufficiently precise to allow extrapolation of the drug effects beyond the treatment duration. Furthermore, our parameterisation may allow further characterisation of PD interactions when evaluating drug combinations (Muliaditan & Della Pasqua, 2020).

The points highlighted above represent an important requirement for the selection of compounds progressing into clinical development. Consequently, we have chosen to explore bacterial growth dynamics in vivo, as in vitro systems do not reproduce the complex environment of the infected host, where a variety of factors may modify the underlying PKPD relationships. These factors include various host defence mechanisms, protein binding, irregular diffusion into healthy, inflamed and infected tissues, and more diverse bacterial heterogeneity (Nuermberger, 2017). In fact, our approach contrasts with the focus of recent initiatives aimed at exploring in vitro systems for compound screening, such as the hollow fibre (HFS‐TB), which offers an invaluable opportunity to explore anti‐tubercular activity without the confounding of physiological barriers, variable distribution due to lesion, granuloma formation, and other physicochemical factors. However, these differences cannot be overlooked when establishing the dose rationale for patients. The relevance of this work is further illustrated by the prediction of the dose range of a novel leucyl‐tRNA synthetase inhibitor (GSK3036656) that produces the highest possible early bactericidal activity in a prospective phase II trial (Tenero et al., 2019).

### 4.3 Potential limitations

We acknowledge that our analysis has various limitations, many of which reflect gaps and deficiencies in current experimental models of TB infection. More extensive prospective validation of the model is clearly warranted. An overview of the limitations of each key assumption in the model as well guidance for model improvements by future work (as more robust experimental data emerges) is therefore presented in Table 2. First, CFU counts represent the total bacterial population, which complicates the identifiability of parameters (e.g., growth and transfer rate constants) describing the growth dynamics. Additional experiments, in which fast‐ and slow‐growing *M. tuberculosis* are measured separately are clearly needed to fully understand the dynamics of both populations over time in mice and confirm the estimated growth rate constant of each population. Second, collection of drug exposure data in individual animals, either in plasma or in lung tissue homogenate should become common practice. Our assumption that PK variability is minor may still hold true, but the availability of individual PK data can improve our understanding of the impact of different sources of variability on the overall anti‐bacterial activity and further increase parameter precision, in particular, potency estimates. Third, a sufficiently wide range of doses is not always available, which results in uncertainty regarding the maximum killing rate.

**TABLE 2:** Overview of the limitations and recommendations for further model improvement

Key assumptions	Limitations and implications	Recommendations for future applications
CFU counts represent the sum of fast‐ and slow‐growing bacterial populations	The growth dynamics model is limited to a two‐state growth condition. The presence of a non‐replicating metabolic phenotype is considered negligible in this infection model. The lack of markers that describe the CFU profiles of each subpopulation makes it difficult to demonstrate experimentally how well the rate parameters (e.g., growth and transfer rate constants) reflect the different growth rates. Consequently, growth rate constants often need to be fixed when the model is applied to new data.	Assess whether the growth dynamics model adequately captures the log and stationary phase in untreated animals: If only total CFU count is available, fix the growth rate constants and recalibrate if needed. If CFU count of each *Mycobacterium tuberculosis* subpopulation is available: re‐estimate growth and transfer rate constants.
Underlying bacterial growth dynamics is best described by the Verhulst model	Description of the stationary phase may not be representative of the CFU profiles for bacterial growth dynamics other than in the investigated murine infection models (i.e., IV, HDA, or LDA).	
Effects of interindividual differences in PK between experiments was assumed to be negligible or minor	Given the lack of individual exposure data, variability in observed anti‐bacterial activity between experiments was attributed to differences in potency. This assumption was based on known differences in bacterial susceptibility and on the effects of changes in the metabolic phenotype during the course of an infection. Estimates of drug potency may be biased due to the exclusion of interindividual variability in PK parameters. Moreover, the lack of individual PK data, makes it difficult to disentangle the drug effect from the contribution of the immune system to the overall antibacterial activity.	Assess whether the model adequately describes the anti‐bacterial activity in the observed data: If concentration versus time profiles are available in individual animals, check whether the PK model can be used to characterise interindividual variability in disposition parameters and refine PK model if needed. If PK data are not available, check whether anti‐bacterial activity can be adequately predicted by the PKPD model and re‐estimate potency if needed.

Abbreviations: HDA, high‐dose aerosol; IV, intravenous; LDA, low‐dose aerosol.

In conclusion, a two‐state growth dynamics model allows the characterisation of CFU counts across different experimental protocols, yielding quantitative estimates of the drug effect on each subpopulation of *M. tuberculosis*. Most importantly, our analysis reveals that bedaquiline is more than 10 times more potent against *M. tuberculosis* than rifampicin. The differences in the potency of bedaquiline and rifampicin on slow‐growing bacteria provide further insight into the requirements for higher rifampicin doses and relatively long treatment periods with drug combinations. We anticipate that such an integrated model will facilitate the ranking of compounds during screening, including improved selection of the doses and partner drugs for combination therapy in patients (Muliaditan & Della Pasqua, 2020).

## AUTHOR CONTRIBUTIONS

M.M. performed the PKPD modelling and data analysis. O.D.P. contributed to the conceptual design, analysis and interpretation of the results. M.M. and O.D.P. wrote the manuscript.

## CONFLICT OF INTEREST

The authors declare no conflict of interest.

## DECLARATION OF TRANSPARENCY AND SCIENTIFIC RIGOUR

This declaration acknowledges that this paper adheres to the principles for transparent reporting and scientific rigour of preclinical research as stated in the *BJP* guidelines for Design and Analysis, and as recommended by funding agencies, publishers and other organisations engaged with supporting research.

## Supporting information


**Figure S1.** Hypothetical time kill curve (dashed line) that was assumed to more closely reflect the maximum killing rate of rifampicin in the Hu experiment. BALB/c mice were infected with H37Rv strain via intravenous infection route and treated with 10–50 mg/kg of rifampicin (5 days/week) for 12 weeks following an incubation period of 2 weeks, as per reported by Hu et al. (Hu, Liu, Ortega‐Muro, Alameda‐Martin, Mitchison & Coates, 2015)). Solid lines represent the corresponding time‐killing curve as published by Hu et al. For the hypothetical maximum drug effect, we assumed that all bacteria were reduced to 10^3.4^ CFU/lung (half of the bacterial load at onset of treatment, 10^6.8^ CFU/lung) after two weeks of treatment with rifampicin and were fully eradicated after three weeks. CFU = colony forming unit
**Figure S2**. Model fit of the Zhang (Zhang, Li & Nuermberger, 2012) experimental data using Gompertz (left panel) or Verhulst growth model (right panel). Growth rates of the slow‐growing population (knetS) was fixed to a range of arbitrary values between 0.0219–6·10–4/h or mean generation time of 1.3–48 days (see Table S1 for further details). Solid lines represent model predictions, whereas open circles represent mean observed data (N = 3 per time point).
**Figure S3**. Results of the sensitivity analysis with Verhulst growth model. Here, the implications of fixing the growth rates of the slow‐growing population (knetS) on the estimated growth rate of the fast‐growing population (knetF) were assessed using experimental data from Zhang et al (Zhang, Li & Nuermberger, 2012). The mean generation time (MGT, in days) is shown above the knetF estimates. Numbers between parentheses on the x‐axis represent the MGT corresponding to the fixed knetS.
**Figure S4**. The proportion of the slow‐growing *Mycobacterium tuberculosis* population at stationary phase corresponding to the various growth rates of the slow‐growing population (knetS) that were initially evaluated. Experimental data from Zhang et al. (Zhang, Li & Nuermberger, 2012) was used for this analysis. Numbers between parentheses on the x‐axis represent the mean generation time (MGT, in days) corresponding to each knetS.
**Figure S5**. Estimated growth rate of the slow growing population (knetS). Here, the implications of assuming different proportions of slow‐growing population at the end of treatment and fixed growth rate of the fastgrowing population (knetF) were assessed using experimental data from Swanson et al (Swanson et al., 2016). Numbers above the open circles represent the mean generation time (MGT) corresponding to the estimated knetS.
**Figure S6**. Model fit (solid lines) of the Swanson et al. data (Swanson et al., 2016) (open circles) given different fixed growth rate for fast growing populations (knetF) and different assumptions on the ratio between fast and slow population at the end of treatment. Each panel is labelled with the assumed percentage of slow growing population at end of treatment, together with the estimated growth rate (knetS).
**Figure S7**. Visual predictive check of the disease model describing the growth dynamics of *M. tuberculosis* in BALB/c mice using experimental data from Hu et al. (left panel) and Gupta et al. (right panel). BALB/c mice were infected with H37Rv strain intravenously or via aerosol route, respectively. Solid lines and shaded area represent the median and 90% prediction interval of the predicted log10 colony forming unit (CFU)/lung over time. Open circles represent the observed log10 CFU/lung.
**Figure S8**. Visual predictive checks (VPC) of the PK models for rifampicin and bedaquiline in mice. Serum PK profiles were derived following single dose administration (bedaquiline) or 10th–13th dose after 2 weeks of daily dosing, 5 days per week (rifampicin). Open circles in the VPC plots represent the mean observed concentrations (3–5 mice per time point). Solid lines and shaded area in the VPCs represent the predicted median and 90% prediction intervals.
**Figure S9**. Model fit of the hypothetical data assumed to more closely represent the maximum killing rate of rifampicin in the Hu experiment (Hu, Liu, Ortega‐Muro, Alameda‐Martin, Mitchison & Coates, 2015). Similar to the experimental protocol used by Hu et al., BALB/c mice were infected via intravenous infection route followed by an incubation period of 2 weeks. To ensure that the simulated rifampicin exposure corresponded to maximum drug effect, the potency against both fast‐ and slow‐growing *M. tuberculosis* (e.g. EC_50_‐F and EC_50_‐S) were empirically fixed to 0.001 mg/L during this estimation step, in conjunction with an artificially high dose treatment (9,999 mg/kg) for 3 weeks (5 days/week). CFU = colony forming unit; PRED = population prediction.
**Table S1**. Comparison of the minimum objective functions yielded by the Verhulst and Gompertz dynamic growth models at various fixed growth rates of the slow‐growing population (knetS).
**Table S2**. Estimated growth rates for fast or slow growing population in the final step.
**Table S3**. Overview of estimated disease parameters for each experiment in the external validation.

## Data Availability

The data that support the findings of this study are available from the corresponding author upon reasonable request.

## References

[bph15688-bib-0001] Alexander, S. P. H. , Kelly, E. , Mathie, A. , Peters, J. A. , Veale, E. L. , Armstrong, J. F. , Faccenda, E. , Harding, S. D. , Pawson, A. J. , Sharman, J. L. , Southan, C. , Buneman, O. P. , Cidlowski, J. A. , Christopoulos, A. , Davenport, A. P. , Fabbro, D. , Spedding, M. , Striessnig, J. , Davies, J. A. , & CGTP Collaborators . (2019). The concise guide to pharmacology 2019/20: Introduction and other protein targets. British Journal of Pharmacology, 176(Suppl 1), S1–S20. 31710719 10.1111/bph.14747 PMC6844537

[bph15688-bib-0002] Alffenaar, J. C. , Gumbo, T. , Dooley, K. E. , Peloquin, C. A. , McIlleron, H. , Zagorski, A. , Cirillo, D. M. , Heysell, S. K. , Silva, D. R. , & Migliori, G. B. (2019). Integrating pharmacokinetics and pharmacodynamics in operational research to end TB. Clinical Infectious Diseases, 70(8), 1774–1780. 10.1093/cid/ciz942 PMC7146003 31560376

[bph15688-bib-0003] Aljayyoussi, G. , Jenkins, V. A. , Sharma, R. , Ardrey, A. , Donnellan, S. , Ward, S. A. , & Biagini, G. A. (2017). Pharmacokinetic‐Pharmacodynamic modelling of intracellular *Mycobacterium tuberculosis* growth and kill rates is predictive of clinical treatment duration. Scientific Reports, 7, 502. 28356552 10.1038/s41598-017-00529-6 PMC5428680

[bph15688-bib-0004] Almeida, D. , Nuermberger, E. , Tasneen, R. , Rosenthal, I. , Tyagi, S. , Williams, K. , Peloquin, C. , & Grosset, J. (2009). Paradoxical effect of isoniazid on the activity of rifampin‐pyrazinamide combination in a mouse model of tuberculosis. Antimicrobial Agents and Chemotherapy, 53, 4178–4184. 19620331 10.1128/AAC.00830-09 PMC2764177

[bph15688-bib-0005] Baron, V. O. , Chen, M. , Clark, S. O. , Williams, A. , Dholakia, K. , & Gillespie, S. H. (2018). Detecting phenotypically resistant *Mycobacterium tuberculosis* using wavelength modulated Raman spectroscopy. Methods in Molecular Biology, 736, 41–50. 10.1007/978-1-4939-7638-6_4 29322457

[bph15688-bib-0006] Bartelink, I. H. , Zhang, N. , Keizer, R. J. , Strydom, N. , Converse, P. J. , Dooley, K. E. , Nuermberger, E. L. , & Savic, R. M. (2017). New paradigm for translational modeling to predict long‐term tuberculosis treatment response. Clinical and Translational Science, 10(5), 366–379. 28561946 10.1111/cts.12472 PMC5593171

[bph15688-bib-0007] Beal, S. , Sheiner, L. B. , Boekmann, A. , & Bauer, R. J. (2009). NONMEM's user's guides. ICON Development Solutions, Ellicott City, Maryland, USA.

[bph15688-bib-0008] Beste, D. J. , Espasa, M. , Bonde, B. , Kierzek, A. M. , Stewart, G. R. , & McFadden, J. (2009). The genetic requirements for fast and slow growth in mycobacteria. PLoS ONE, 4, e5349. 19479006 10.1371/journal.pone.0005349 PMC2685279

[bph15688-bib-0009] Chen, C. , Ortega, F. , Rullas, J. , Alameda, L. , Angulo‐Barturen, I. , Ferrer, S. , & Simonsson, U. S. H. (2017). The multistate tuberculosis pharmacometric model: A semi‐mechanistic pharmacokinetic‐pharmacodynamic model for studying drug effects in an acute tuberculosis mouse model. Journal of Pharmacokinetics and Pharmacodynamics, 44(2), 133–141. 28205025 10.1007/s10928-017-9508-2 PMC5376397

[bph15688-bib-0010] Chen, C. , Wicha, S. G. , de Knegt, G. J. , Ortega, F. , Alameda, L. , Sousa, V. , de Steenwinkel, J. E. M. , & Simonsson, U. S. H. (2017). Assessing pharmacodynamic interactions in mice using the multistate tuberculosis pharmacometric and general pharmacodynamic interaction models. CPT: Pharmacometrics & Systems Pharmacology, 6(11), 787–797. 28657202 10.1002/psp4.12226 PMC5702905

[bph15688-bib-0011] Clewe, O. , Aulin, L. , Hu, Y. , Coates, A. R. , & Simonsson, U. S. (2016). A multistate tuberculosis pharmacometric model: A framework for studying anti‐tubercular drug effects in vitro. The Journal of Antimicrobial Chemotherapy, 71, 964–974. 26702921 10.1093/jac/dkv416 PMC4790616

[bph15688-bib-0012] De Groote, M. A. , Gilliland, J. C. , Wells, C. L. , Brooks, E. J. , Woolhiser, L. K. , Gruppo, V. , Peloquin, C. A. , Orme, I. M. , & Lenaerts, A. J. (2011). Comparative studies evaluating mouse models used for efficacy testing of experimental drugs against *Mycobacterium tuberculosis* . Antimicrobial Agents and Chemotherapy, 55, 1237–1247. 21135176 10.1128/AAC.00595-10 PMC3067068

[bph15688-bib-0013] de Steenwinkel, J. E. , Aarnoutse, R. E. , de Knegt, G. J. , ten Kate, M. T. , Teulen, M. , Verbrugh, H. A. , Boeree, M. J. , van Soolingen, D. , & Bakker‐Woudenberg, I. A. (2013). Optimization of the rifampin dosage to improve the therapeutic efficacy in tuberculosis treatment using a murine model. American Journal of Respiratory and Critical Care Medicine, 187, 1127–1134. 23525933 10.1164/rccm.201207-1210OC

[bph15688-bib-0014] de Steenwinkel, J. E. , de Knegt, G. J. , ten Kate, M. T. , van Belkum, A. , Verbrugh, H. A. , Kremer, K. , van Soolingen, D. , & Bakker‐Woudenberg, I. A. (2010). Time‐kill kinetics of anti‐tuberculosis drugs, and emergence of resistance, in relation to metabolic activity of *Mycobacterium tuberculosis* . The Journal of Antimicrobial Chemotherapy, 65, 2582–2589. 20947621 10.1093/jac/dkq374

[bph15688-bib-0015] de Steenwinkel, J. E. , ten Kate, M. T. , de Knegt, G. J. , Verbrugh, H. A. , van Belkum, A. , Hernandez‐Pando, R. , & Bakker‐Woudenberg, I. A. (2011). Course of murine tuberculosis and response to first‐line therapy depends on route of infection and inoculum size. The International Journal of Tuberculosis and Lung Disease, 15, 1478–1484. 22008760 10.5588/ijtld.11.0012

[bph15688-bib-0016] Dhillon, J. , Andries, K. , Phillips, P. P. , & Mitchison, D. A. (2010). Bactericidal activity of the diarylquinoline TMC207 against mycobacterium tuberculosis outside and within cells. Tuberculosis (Edinburgh, Scotland), 90, 301–305. 10.1016/j.tube.2010.07.004 20732832

[bph15688-bib-0017] Driver, E. R. , Ryan, G. J. , Hoff, D. R. , Irwin, S. M. , Basaraba, R. J. , Kramnik, I. , & Lenaerts, A. J. (2012). Evaluation of a mouse model of necrotic granuloma formation using C3HeB/FeJ mice for testing of drugs against *Mycobacterium tuberculosis* . Antimicrobial Agents and Chemotherapy, 56, 3181–3195. 22470120 10.1128/AAC.00217-12 PMC3370740

[bph15688-bib-0018] Franzblau, S. G. , DeGroote, M. A. , Cho, S. H. , Andries, K. , Nuermberger, E. , Orme, I. M. , Mdluli, K. , Angulo‐Barturen, I. , Dick, T. , Dartois, V. , & Lenaerts, A. J. (2012). Comprehensive analysis of methods used for the evaluation of compounds against *Mycobacterium tuberculosis* . Tuberculosis (Edinburgh, Scotland), 92, 453–488. 10.1016/j.tube.2012.07.003 22940006

[bph15688-bib-0019] Garrett, E. R. (1978). Kinetics of antimicrobial action. Scandinavian Journal of Infectious Diseases. Supplementum, 14, 54–85. PMC8334812 30169

[bph15688-bib-0020] Gumbo, T. , Angulo‐Barturen, I. , & Ferrer‐Bazaga, S. (2015). Pharmacokinetic‐pharmacodynamic and dose‐response relationships of antituberculosis drugs: Recommendations and standards for industry and academia. The Journal of Infectious Diseases, 211(Suppl 3), S96–S106. 26009618 10.1093/infdis/jiu610

[bph15688-bib-0021] Gupta, S. , Tyagi, S. , & Bishai, W. R. (2015). Verapamil increases the bactericidal activity of bedaquiline against *Mycobacterium tuberculosis* in a mouse model. Antimicrobial Agents and Chemotherapy, 59, 673–676. 25331694 10.1128/AAC.04019-14 PMC4291418

[bph15688-bib-0022] Hammond, R. J. , Baron, V. O. , Oravcova, K. , Lipworth, S. , & Gillespie, S. H. (2015). Phenotypic resistance in mycobacteria: Is it because I am old or fat that I resist you? The Journal of Antimicrobial Chemotherapy, 70, 2823–2827. 26163401 10.1093/jac/dkv178

[bph15688-bib-0023] Hammond, R. J. H. , Baron, V. O. , Lipworth, S. , & Gillespie, S. H. (2018). Enhanced methodologies for detecting phenotypic resistance in mycobacteria. Methods in Molecular Biology, 1736, 85–89. 29322461 10.1007/978-1-4939-7638-6_8

[bph15688-bib-0024] Harding, S. D. , Sharman, J. L. , Faccenda, E. , Southan, C. , Pawson, A. J. , Ireland, S. , Gray, A. J. G. , Bruce, L. , Alexander, S. P. H. , Anderton, S. , Bryant, C. , Davenport, A. P. , Doerig, C. , Fabbro, D. , Levi‐Schaffer, F. , Spedding, M. , Davies, J. A. , & NC‐IUPHAR . (2018). The IUPHAR/BPS guide to pharmacology 2018: Updates and expansion to encompass the new guide to immunopharmacology. Nucleic Acids Research, 46, D1091–D1106. 29149325 10.1093/nar/gkx1121 PMC5753190

[bph15688-bib-0025] Hoff, D. R. , Ryan, G. J. , Driver, E. R. , Ssemakulu, C. C. , De Groote, M. A. , Basaraba, R. J. , & Lenaerts, A. J. (2011). Location of intra‐ and extracellular M. tuberculosis populations in lungs of mice and guinea pigs during disease progression and after drug treatment. PLoS ONE, 6, e17550. 21445321 10.1371/journal.pone.0017550 PMC3061964

[bph15688-bib-0026] Hu, Y. , Liu, A. , Ortega‐Muro, F. , Alameda‐Martin, L. , Mitchison, D. , & Coates, A. (2015). High‐dose rifampicin kills persisters, shortens treatment duration, and reduces relapse rate in vitro and in vivo. Frontiers in Microbiology, 6, 641. 26157437 10.3389/fmicb.2015.00641 PMC4477163

[bph15688-bib-0027] Hu, Y. , Pertinez, H. , Ortega‐Muro, F. , Alameda‐Martin, L. , Liu, Y. , Schipani, A. , Davies, G. , & Coates, A. (2016). Investigation of elimination rate, persistent subpopulation removal, and relapse rates of *Mycobacterium tuberculosis* by using combinations of first‐line drugs in a modified Cornell mouse model. Antimicrobial Agents and Chemotherapy, 60, 4778–4785. 27216065 10.1128/AAC.02548-15 PMC4958161

[bph15688-bib-0028] Irwin, S. M. , Prideaux, B. , Lyon, E. R. , Zimmerman, M. D. , Brooks, E. J. , Schrupp, C. A. , Chen, C. , Reichlen, M. J. , Asay, B. C. , Voskuil, M. I. , Nuermberger, E. L. , Andries, K. , Lyons, M. A. , Dartois, V. , & Lenaerts, A. J. (2016). Bedaquiline and pyrazinamide treatment responses are affected by pulmonary lesion heterogeneity in *Mycobacterium tuberculosis* infected C3HeB/FeJ mice. ACS Infectious Diseases, 2, 251–267. 27227164 10.1021/acsinfecdis.5b00127 PMC4874602

[bph15688-bib-0029] Lipworth, S. , Hammond, R. J. H. , Baron, V. O. , Hu, Y. , Coates, A. , & Gillespie, S. H. (2016). Defining dormancy in mycobacterial disease. Tuberculosis, 99, 131–142. 27450015 10.1016/j.tube.2016.05.006

[bph15688-bib-0030] Mitchison, D. A. (1985). The action of antituberculosis drugs in short‐course chemotherapy. Tubercle, 66, 219–225. 3931319 10.1016/0041-3879(85)90040-6

[bph15688-bib-0031] Muliaditan, M. , Davies, G. R. , Simonsson, U. S. , Gillespie, S. H. , & Della Pasqua, O. (2017). The implications of model‐informed drug discovery and development for tuberculosis. Drug Discovery Today, 22, 481–486. 27693714 10.1016/j.drudis.2016.09.004

[bph15688-bib-0032] Muliaditan, M. , & Della Pasqua, O. (2020). Evaluation of pharmacokinetic‐pharmacodynamic relationships and selection of drug combinations for tuberculosis. British Journal of Clinical Pharmacology, 87, 140–151. 32415743 10.1111/bcp.14371

[bph15688-bib-0033] Myers, A. J. , Mariano, S. , Kirschner, D. E. , & Flynn, J. L. (2013). Inoculation dose of *Mycobacterium tuberculosis* does not influence priming of T cell responses in lymph nodes. Journal of Immunology, 190, 4707–4716. 10.4049/jimmunol.1203465 PMC3674545 23547119

[bph15688-bib-0034] Nielsen, E. I. , Cars, O. , & Friberg, L. E. (2011). Predicting in vitro antibacterial efficacy across experimental designs with a semimechanistic pharmacokinetic‐pharmacodynamic model. Antimicrobial Agents and Chemotherapy, 55, 1571–1579. 21282424 10.1128/AAC.01286-10 PMC3067154

[bph15688-bib-0035] Nuermberger, E. L. (2017). Preclinical efficacy testing of new drug candidates. Microbiology Spectrum, 5(3), TBTB2‐0034‐2017. 10.1128/microbiolspec.tbtb2-0034-2017 PMC11687513 28643624

[bph15688-bib-0036] Peleg, M. , & Corradini, M. G. (2011). Microbial growth curves: What the models tell us and what they cannot. Critical Reviews in Food Science and Nutrition, 51, 917–945. 21955092 10.1080/10408398.2011.570463

[bph15688-bib-0037] R Core Team . (2016). R: A language and environment for statistical computing. R Foundation for Statistical Computing. https://www.R-project.org/

[bph15688-bib-0038] Raffetseder, J. , Pienaar, E. , Blomgran, R. , Eklund, D. , Patcha Brodin, V. , Andersson, H. , Welin, A. , & Lerm, M. (2014). Replication rates of *Mycobacterium tuberculosis* in human macrophages do not correlate with mycobacterial antibiotic susceptibility. PLoS ONE, 9, e112426. 25386849 10.1371/journal.pone.0112426 PMC4227709

[bph15688-bib-0039] Rohatgi, A. (2017). WebPlotDigitizer. http://arohatgi.info/WebPlotDigitizer

[bph15688-bib-0040] Rosenthal, I. M. , Tasneen, R. , Peloquin, C. A. , Zhang, M. , Almeida, D. , Mdluli, K. E. , Karakousis, P. C. , Grosset, J. H. , & Nuermberger, E. L. (2012). Dose‐ranging comparison of rifampin and rifapentine in two pathologically distinct murine models of tuberculosis. Antimicrobial Agents and Chemotherapy, 56, 4331–4340. 22664964 10.1128/AAC.00912-12 PMC3421552

[bph15688-bib-0041] Sheiner, L. B. (1997). Learning versus confirming in clinical drug development. Clinical Pharmacology and Therapeutics, 61, 275–291. 9084453 10.1016/S0009-9236(97)90160-0

[bph15688-bib-0042] Sloan, D. J. , Mwandumba, H. C. , Garton, N. J. , Khoo, S. H. , Butterworth, A. E. , Allain, T. J. , Heyderman, R. S. , Corbett, E. L. , Barer, M. R. , & Davies, G. R. (2015). Pharmacodynamic modeling of bacillary elimination rates and detection of bacterial lipid bodies in sputum to predict and understand outcomes in treatment of pulmonary tuberculosis. Clinical Infectious Diseases, 61, 1–8. 25778753 10.1093/cid/civ195 PMC4463005

[bph15688-bib-0043] Swanson, R. V. , Ammerman, N. C. , Ngcobo, B. , Adamson, J. , Moodley, C. , Dorasamy, A. , Moodley, S. , Mgaga, Z. , Bester, L. A. , Singh, S. D. , Almeida, D. V. , & Grosset, J. H. (2016). Clofazimine contributes to sustained antimicrobial activity after treatment cessation in a mouse model of tuberculosis chemotherapy. Antimicrobial Agents and Chemotherapy, 60, 2864–2869. 26926638 10.1128/AAC.00177-16 PMC4862514

[bph15688-bib-0044] Tasneen, R. , Betoudji, F. , Tyagi, S. , Li, S. Y. , Williams, K. , Converse, P. J. , Dartois, V. , Yang, T. , Mendel, C. M. , Mdluli, K. E. , & Nuermberger, E. L. (2015). Contribution of oxazolidinones to the efficacy of novel regimens containing bedaquiline and pretomanid in a mouse model of tuberculosis. Antimicrobial Agents and Chemotherapy, 60, 270–277. 26503656 10.1128/AAC.01691-15 PMC4704221

[bph15688-bib-0045] Tasneen, R. , Li, S. Y. , Peloquin, C. A. , Taylor, D. , Williams, K. N. , Andries, K. , Mdluli, K. E. , & Nuermberger, E. L. (2011). Sterilizing activity of novel TMC207‐ and PA‐824‐containing regimens in a murine model of tuberculosis. Antimicrobial Agents and Chemotherapy, 55, 5485–5492. 21930883 10.1128/AAC.05293-11 PMC3232786

[bph15688-bib-0046] Tasneen, R. , Tyagi, S. , Williams, K. , Grosset, J. , & Nuermberger, E. (2008). Enhanced bactericidal activity of rifampin and/or pyrazinamide when combined with PA‐824 in a murine model of tuberculosis. Antimicrobial Agents and Chemotherapy, 52, 3664–3668. 18694943 10.1128/AAC.00686-08 PMC2565869

[bph15688-bib-0047] Tenero, D. , Derimanov, G. , Carlton, A. , Tonkyn, J. , Davies, M. , Cozens, S. , Gresham, S. , Gaudion, A. , Puri, A. , Muliaditan, M. , & Rullas‐Trincado, J. (2019). First‐time‐in‐human study and prediction of early bactericidal activity for GSK3036656, a potent leucyl‐tRNA synthetase inhibitor for tuberculosis treatment. Antimicrobial Agents and Chemotherapy, 63, e00240–19. 31182528 10.1128/AAC.00240-19 PMC6658769

[bph15688-bib-0048] van Heeswijk, R. P. , Dannemann, B. , & Hoetelmans, R. M. (2014). Bedaquiline: A review of human pharmacokinetics and drug‐drug interactions. The Journal of Antimicrobial Chemotherapy, 69, 2310–2318. 24860154 10.1093/jac/dku171

[bph15688-bib-0049] van Ingen, J. , Aarnoutse, R. E. , Donald, P. R. , Diacon, A. H. , Dawson, R. , Plemper van Balen, G. , Gillespie, S. H. , & Boeree, M. J. (2011). Why do we use 600 mg of rifampicin in tuberculosis treatment? Clinical Infectious Diseases, 52, e194–e199. 21467012 10.1093/cid/cir184

[bph15688-bib-0050] Vijay, S. , Nair, R. R. , Sharan, D. , Jakkala, K. , Mukkayyan, N. , Swaminath, S. , Pradhan, A. , Joshi, N. V. , & Ajitkumar, P. (2019). Mycobacterial cultures contain cell size and density specific sub‐populations of cells with significant differential susceptibility to antibiotics, oxidative and nitrite stress. Frontiers in Microbiology, 8, 463. 10.3389/fmicb.2017.00463 PMC5359288 28377757

[bph15688-bib-0051] Warner, D. F. , & Mizrahi, V. (2014). Shortening treatment for tuberculosis—To basics. The New England Journal of Medicine, 371, 1642–1643. 25337754 10.1056/NEJMe1410977

[bph15688-bib-0052] Zhang, T. , Li, S. Y. , & Nuermberger, E. L. (2012). Autoluminescent *Mycobacterium tuberculosis* for rapid, real‐time, non‐invasive assessment of drug and vaccine efficacy. PLoS ONE, 7, e29774. 22253776 10.1371/journal.pone.0029774 PMC3256174

