# Supplementary material for: Bacterial growth dynamics and pharmacokinetic–pharmacodynamic relationships of rifampicin and bedaquiline in BALB/c mice
Source: Br J Pharmacol. 2021 Dec 27;179(6):1251–63. doi: 10.1111/bph.15688 (PMC9303191; doi:10.1111/bph.15688)
Supplement: Supplementary file 1 — Figure S1. Hypothetical time kill curve (dashed line) that was assumed to more closely reflect the maximum killing rate of rifampicin in the Hu experiment. BALB/c mice were infected with H37Rv strain via intravenous infection route and treated with 10–50 mg/kg of rifampicin (5 days/week) for 12 weeks following an incubation period of 2 weeks, as per reported by Hu et al. (Hu, Liu, Ortega‐Muro, Alameda‐Martin, Mitchison & Coates, 2015)). Solid lines represent the corresponding time‐killing curve as published by Hu et al. For the hypothetical maximum drug effect, we assumed that all bacteria were reduced to 103.4 CFU/lung (half of the bacterial load at onset of treatment, 106.8 CFU/lung) after two weeks of treatment with rifampicin and were fully eradicated after three weeks. CFU = colony forming unit Figure S2. Model fit of the Zhang (Zhang, Li & Nuermberger, 2012) experimental data using Gompertz (left panel) or Verhulst growth model (right panel). Growth rates of the slow‐growing population (knetS) was fixed to a range of arbitrary values between 0.0219–6·10–4/h or mean generation time of 1.3–48 days (see Table S1 for further details). Solid lines represent model predictions, whereas open circles represent mean observed data (N = 3 per time point). Figure S3. Results of the sensitivity analysis with Verhulst growth model. Here, the implications of fixing the growth rates of the slow‐growing population (knetS) on the estimated growth rate of the fast‐growing population (knetF) were assessed using experimental data from Zhang et al (Zhang, Li & Nuermberger, 2012). The mean generation time (MGT, in days) is shown above the knetF estimates. Numbers between parentheses on the x‐axis represent the MGT corresponding to the fixed knetS. Figure S4. The proportion of the slow‐growing Mycobacterium tuberculosis population at stationary phase corresponding to the various growth rates of the slow‐growing population (knetS) that were initially evaluated. Experimental data from Z [file BPH-179-1251-s001.pdf]

Supporting Information

Table of Contents

Methods 2

Step 1: estimation of the growth rate of fast-growing *M. tuberculosis* population 2

Step 2: estimation of the growth rate of the slow-growing *M. tuberculosis* population 2

Step 3: selection of growth rates for fast- and slow-growing *M. tuberculosis* 3

Population pharmacokinetic analysis of rifampicin and bedaquiline in mice 5

Results 6

Step 1: estimation of the growth rate of fast-growing *M. tuberculosis* population 6

Step 2: estimation of the growth rate of the slow-growing *M. tuberculosis* population 8

Step 3: selection of growth rates for fast and slow-growing *M. tuberculosis* 11

References 16

Methods

Step 1: estimation of the growth rate of fast-growing M. tuberculosis population

Data from Zhang *et al.* was used for this purpose. We have assumed that all *M. tuberculosis* are in the F state at the onset of infection. A range between 0.03/h to 0.004/h (i.e. MGT between 21 **(Aljayyoussi et al., 2017)** and 158 hours **(Raffetseder et al., 2014)** has been reported in literature with regard to *in vitro* growth rates of slow-growing *M. tuberculosis*. Yet, given that no consensus could be found regarding the growth rate of slow-growing bacteria *in vivo*, parameter estimation was performed under the assumption that knetS was unknown.

A sensitivity analysis was therefore performed to evaluate the estimates of knetF and predicted ratio between F and S population over time, when knetS is fixed to a range of arbitrary values between 0.0219 and 6·10-4/h (i.e. MGT between 1.3 and 48.1 days). The three most plausible knetF estimates (i.e. measured by lowest MVOF and model fit) were then selected for the next step.

Step 2: estimation of the growth rate of the slow-growing M. tuberculosis population

Data from Swanson *et al*. was used for this evaluation, during which we aimed to identify knetS whilst fixing knetF to the range of values estimated in step 1. The main assumption in this step was that only the S population was present in lung tissue after the 8 weeks of treatment with first-line drugs (i.e., the regrowth phase started from the remaining S population). To validate this assumption, we also explored the implications of different experimental conditions, such as different ratio of F:S at the onset of regrowth on the estimated knetS, including 50-50, 40-60, 30-70, 20-80, 10-90 and 0-100. MVOF values and standard model diagnostic criteria were used to assess the goodness-of-fit and biological plausibility of the estimated values. The three best knetS were selected for the final step of the model building.

Step 3: selection of growth rates for fast and slow-growing M. tuberculosis

In this final step, we aimed to establish which of the estimated ranges of knetF and knetS could best describe the available experimental data from Swanson *et al.* (Swanson et al., 2016) and Zhang *et al.* (Zhang, Li & Nuermberger, 2012). The Zhang data set was used once more to estimate knetF while knetS was fixed to the estimates from Step 2. In parallel, knetS was also estimated using the Swanson data set, while knetF was fixed to the estimates from Step 1. The combination of knetF and knetS values obtained from each data set separately, was subsequently selected for the final parameterisation of the growth dynamics model.

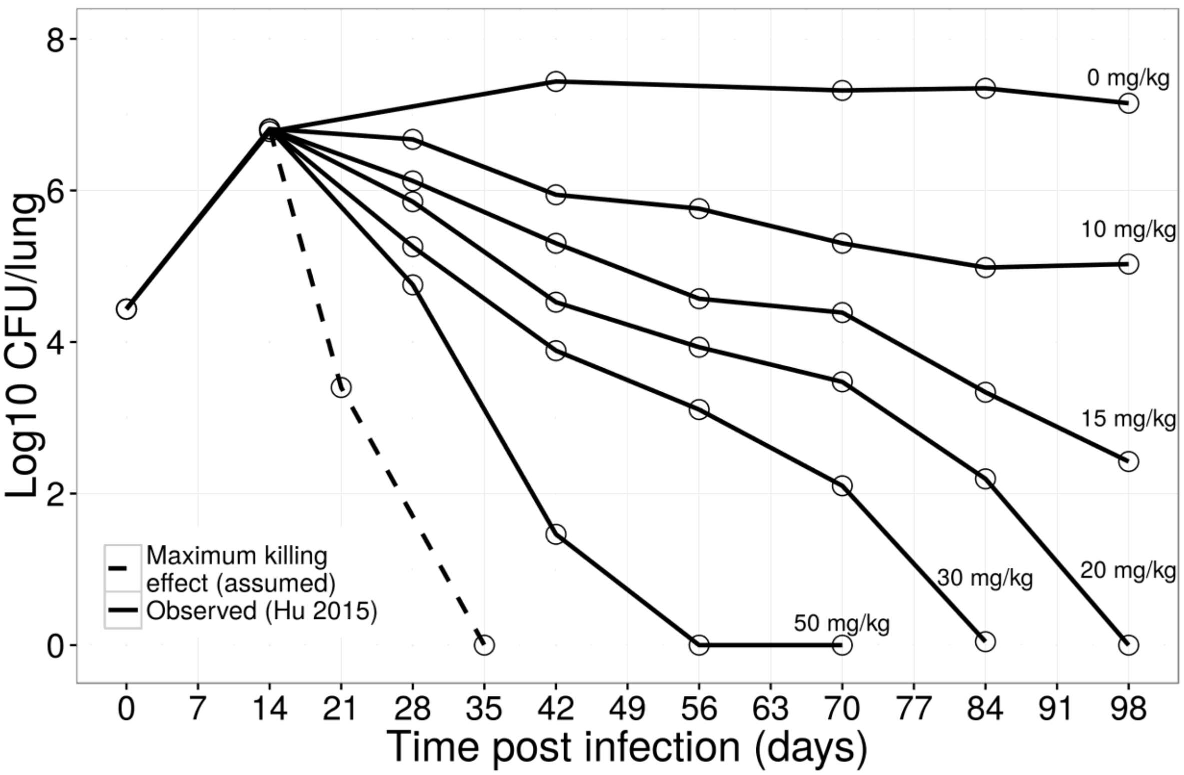

Figure S1. Hypothetical time kill curve (dashed line) that was assumed to best reflect the maximum killing rate of rifampicin in the experiment performed by Hu et al. (Hu, Liu, Ortega-Muro, Alameda-Martin, Mitchison & Coates, 2015). In their protocol, BALB/c mice were infected with H37Rv strain via intravenous infection route and treated with 10-50 mg/kg of rifampicin (5 days/week) for 12 weeks following an incubation period of 2 weeks. Solid lines represent the corresponding time-killing curve as published by Hu *et al*. For the hypothetical maximum drug effect, we assumed that all bacteria were reduced to $10^{3.4}$ CFU/lung (half of the bacterial load at onset of treatment, $10^{6.8}$ CFU/lung) after one week of treatment with rifampicin and were fully eradicated after three weeks. CFU = colony forming unit.

Population pharmacokinetics of rifampicin and bedaquiline in mice

Population pharmacokinetic modelling was based on a nonlinear mixed effects approach using ADVAN6 TRANS1 subroutines and first-order conditional estimation method with interaction (FOCE-I) in NONMEM 7.3. Various compartmental PK models with first-order absorption and elimination were evaluated and selected based on goodness-of-fit criteria. Whilst a nonlinear mixed effects model allows the characterisation of inter-individual variability, this was not possible during model building and evaluation, as published data consisted of mean concentration vs. time profiles only. Given the homogeneity of the data and limited variability in the body weight of the mice, no covariates were explored. Consequently, only residual variability was estimated. The residual variability associated with mean concentrations was described according to an exponential model (equation 1).

$$\mathrm{Y} = \mathrm{PRED} \cdot \mathrm{EXP}(\mathrm{EPS}(1)) \quad (1)$$

where *PRED* is the population predicted concentration and *EPS* the residual variability, which is assumed to follow a normal distribution with mean 0 and variance σ^2.

Goodness-of-fit was assessed by statistical and graphical methods, including population predicted *vs.* observed concentrations, conditional weighted residuals (CWRES) *vs.* observed concentrations and time. Comparison of models was based on the changes in the minimum value of objective function (ΔMVOF). Given that ΔMVOF is approximately χ^2 distributed, a ΔMVOF of ≥3.84 corresponds to a significance level of ≤0.05 for 1 degree of freedom (df). Visual predictive checks (VPC; 1000 iterations) were used for final model evaluation. Variation in the predicted concentrations was generated from residual error only.

Results

Selection of system-specific parameters in the bacterial growth dynamics model

Step 1: estimation of the growth rate of fast-growing M. tuberculosis population

Table S1. Comparison of the minimum objective function values yielded by the Verhulst and Gompertz dynamic growth models at various fixed growth rates of the slow-growing population (knetS).

Fixed knetS (h^{-1}) [mean generation time in days]	Minimum value of objective function (MVOF)		ΔMVOF
	Verhulst (reference)	Gompertz	
0.0219 [1.3]	-5.065	6.846	11.911*
0.011 [2.6]	-4.816	-3.328	1.488
0.01 [2.9]	-4.779	-3.795	0.984
0.0073 [4]	-4.652	-0.303	4.349*
0.0055 [5.3]	-4.533	0.581	5.114*
0.0037 [7.8]	-4.376	0.701	5.077*
0.0029 [10]	-4.296	0.72	5.016*
0.0022 [13.1]	-4.234	0.713	4.947*
0.0015 [19.3]	-4.211	0.678	4.889*
0.0011 [26.3]	-4.245	0.642	4.887*
7E-04 [41.3]	-4.351	0.591	4.942*
6E-04 [48.1]	-4.395	0.576	4.971*
** p. value < 0.05 as indicated by a ΔMVOF of >3.84.*			

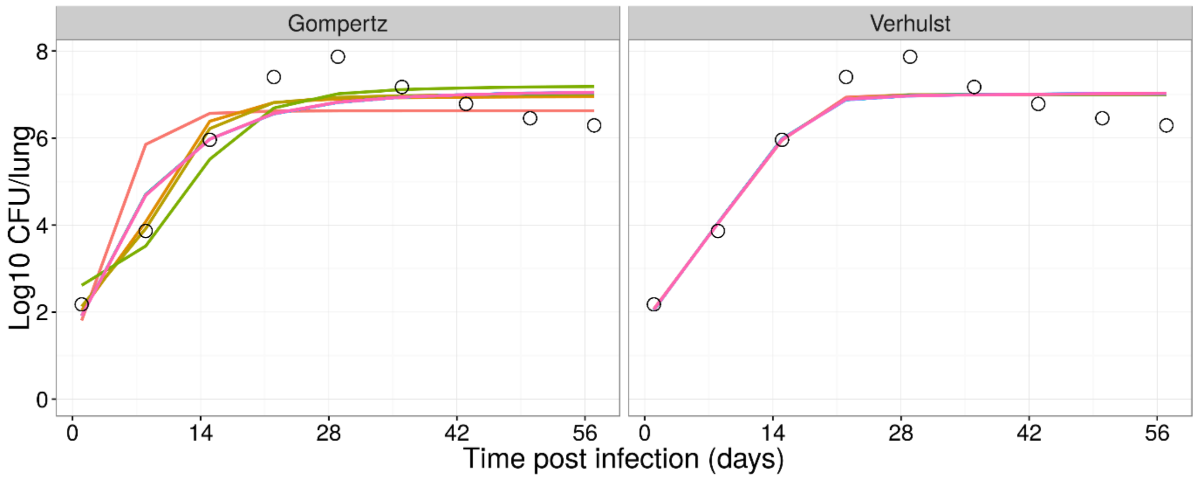

Figure S2. Model fit of the experimental data from Zhang et al. (Zhang, Li & Nuermberger, 2012) using the Gompertz (left panel) or Verhulst growth model (right panel). Growth rate constant for the slow-growing population (knetS) was fixed to a range of arbitrary values between 0.0219-6·10-4/h or mean generation time of 1.3-48 days (see **Table S1** for further details). Solid lines represent model predictions, whereas open circles depict the mean observed data (N=3 per time point).

In the subsequent steps during model building, we have found that despite 36.5-fold change in fixed growth rate for the slow population, estimated knetF values ranged minimally from 0.0269 to 0.0274/h (**Figure S3**). The predicted proportion of the S population was correlated to knetS (**Figure S4**). For example, when knetS was fixed to 0.0219/h, the model predicted that 55% of the total population at stationary phase would consist of S, while a knetS of $6 \cdot 10^{-4}$/h was associated with 97.6% of the population being in the S state. This can be explained by the transfer rate constants kFS and kSF in the model, which are affected by the growth rate constants (i.e., knetF and knetS). knetS values between 0.271 and 0.273/h (each corresponding to MGT of approximately 25 hours) were selected for the subsequent steps of the analysis.

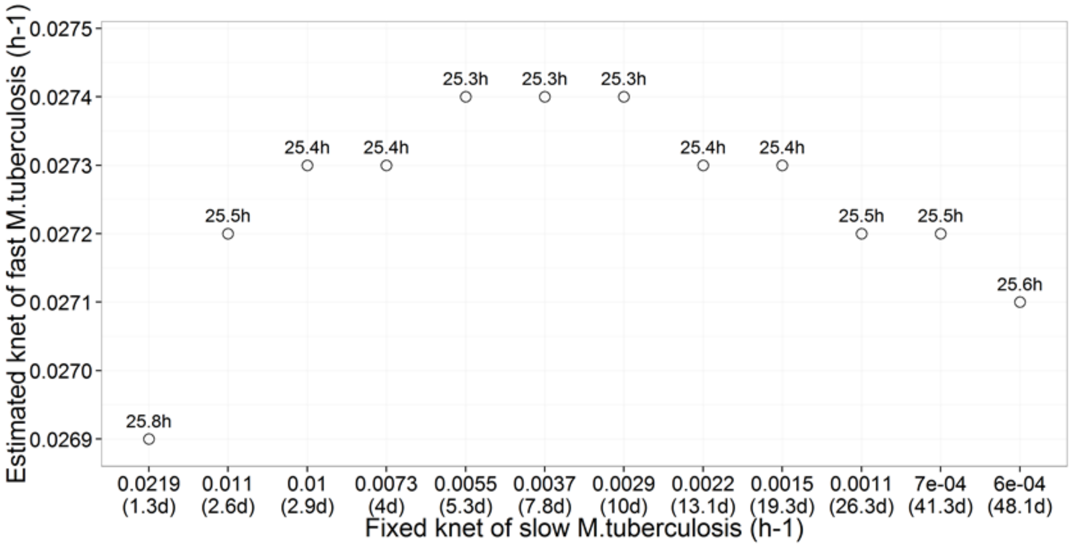

Figure S3. Results of the sensitivity analysis with the Verhulst growth model. Here, the implications of fixing the growth rate constant of the slow-growing population (knetS) on the estimated growth rate constant of the fast-growing population (knetF) were assessed using experimental data from Zhang *et al* (Zhang, Li & Nuermberger, 2012). The mean generation time (MGT, in days) is shown above the knetF estimates. Numbers between parentheses on the x-axis represent the MGT corresponding to the fixed knetS.

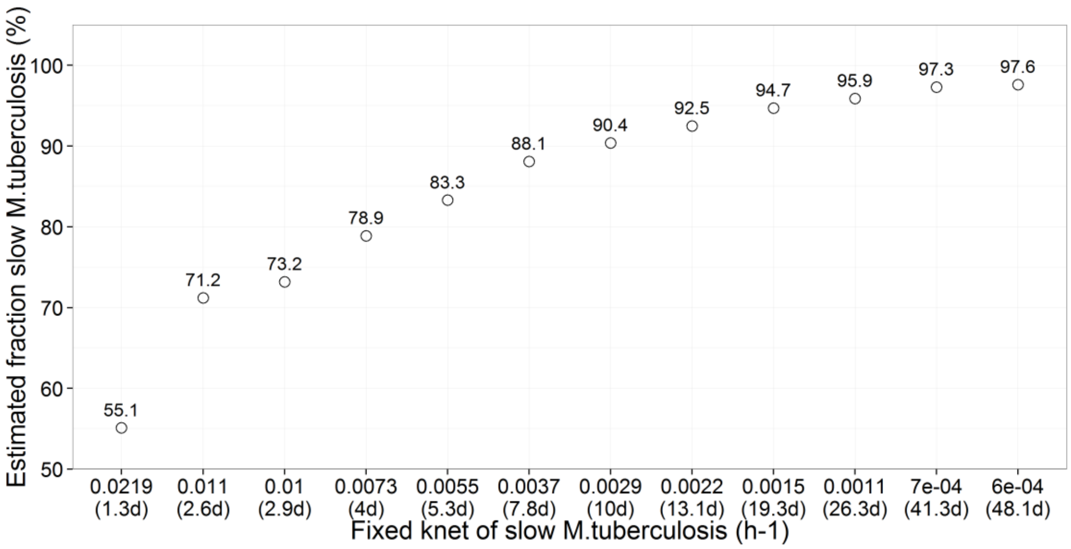

Figure S4. The proportion of the slow-growing *M. tuberculosis* population at stationary phase corresponding to the various growth rate constant values of the slow-growing population (knetS) that were initially evaluated. Experimental data from Zhang *et al.* (Zhang, Li & Nuermberger, 2012) were used for this analysis. Numbers between parentheses on the x-axis represent the mean generation time (MGT, in days) corresponding to each knetS.

Step 2: estimation of the growth rate of the slow-growing M. tuberculosis population

Similarly, the growth rate of the slow growing population was evaluated using data from Swanson *et al.* **(Swanson et al., 2016)**. **Figure S5** shows that knetF values (0.271-0.273/h) always yielded comparable knetS (0.00067-0.00069/h; MGT of 42-43 days). With respect to whether the F population should be assumed to be present at the end of treatment period, **Figure S6** clearly shows that the Swanson data was poorly described when such assumption is made. This result provided support for our initial hypothesis that only the S population survived following the 8-week treatment period with the first line drugs.

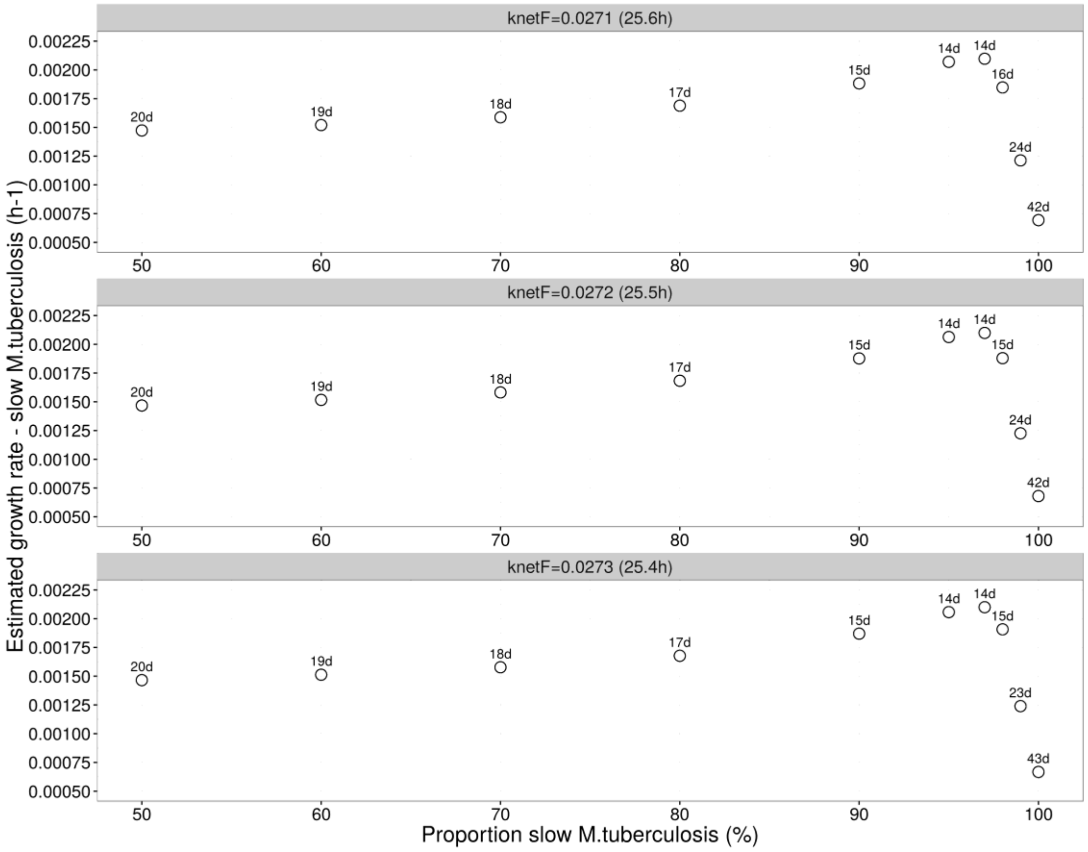

Figure S5. Estimated growth rate constant of the slow-growing population (knetS). Here, the implications of assuming different proportions of the slow-growing population at the end of treatment and fixed growth rate constant of the fast-growing population (knetF) were assessed using the experimental data from Swanson *et al* (Swanson et al., 2016). Numbers above the open circles represent the mean generation time (MGT) corresponding to the estimated knetS.

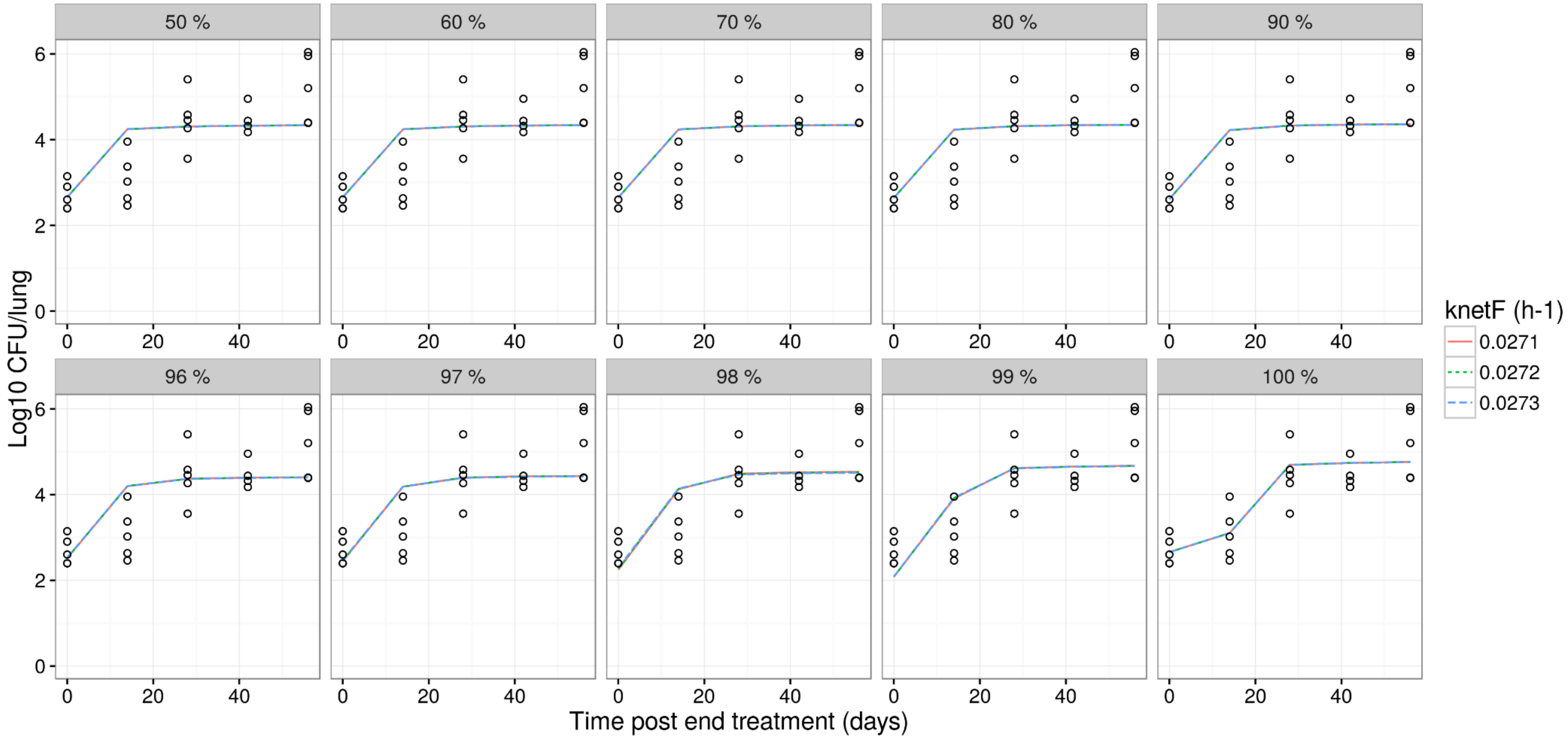

Figure S6. Model fit (solid lines) of the Swanson *et al.* data (Swanson et al., 2016) (open circles) after fixing different growth rate constant values for the fast-growing population (knetF) and different assumptions on the ratio between fast- and slow-growing population at the end of treatment. Each panel is labelled with the assumed percentage of slow-growing population at end of treatment, together with the estimated growth rate constant (knetS).

Step 3: selection of growth rates for fast and slow-growing M. tuberculosis

Here we explored which set of parameter estimates for knetF and knetS best described both the experimental data from Swanson *et al.* (Swanson et al., 2016) and Zhang *et al.* (Zhang, Li & Nuermberger, 2012). Growth rate constants for the F population were estimated using the data set from Zhang *et al*. knetS was fixed to the following values: 0.00067/h, 0.00068/h and 0.00069/h (MGT of 42-43 days; estimated from Step 2, as described previously). In parallel, growth rate constants for the S population were estimated using the data set from Swanson *et al*. knetF was fixed to the following values: 0.0271/h, 0.0272/h and 0.0273/h (MGT of 25-26 hours; estimated as outlined in Step 1).

As shown in **Table S2**, knetF values remained at 0.0272/h, irrespective of the choice of knetS. The combination of knetF of 0.0272/h and knetS of 0.00068/h was identified as the final estimates describing the growth rates of the two subpopulations. These values corresponded to an MGT of respectively 25 and 1019 h (or 42 days).

Table S2. Estimated growth rate constants for fast- and slow-growing populations in the final step.

**Data set**	**Growth rate constant of fast-growing population, knetF (h-1)**	**Growth rate constant of slow-growing population, knetS (h-1)**
Zhang *et al.*, 2012	0.0272	0.00067 FIX
	**0.0272***	**0.00068 FIX**
	0.0272	0.00069 FIX
Swanson *et al.*, 2016	0.0271 FIX	0.00069
	**0.0272 FIX**	**0.00068***
	0.0273 FIX	0.00067
** Selected as the final parameter values for knetF and knetS in the growth dynamics model.*		

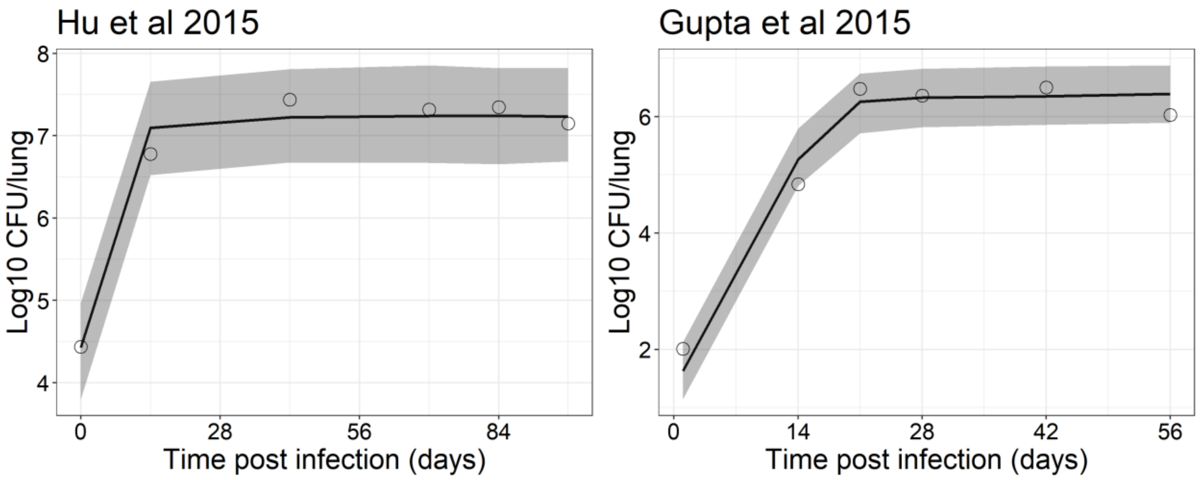

Figure S7. Visual predictive check of the model describing the growth dynamics of *M. tuberculosis* in BALB/c mice using the experimental data from Hu et al. (left panel) and Gupta et al. (right panel). BALB/c mice were infected with H37Rv strain intravenously or via aerosol route, respectively. Solid lines and shaded area represent the median and 90% prediction interval of the predicted log10 colony forming unit (CFU)/lung over time. Open circles represent the observed log10 CFU/lung.

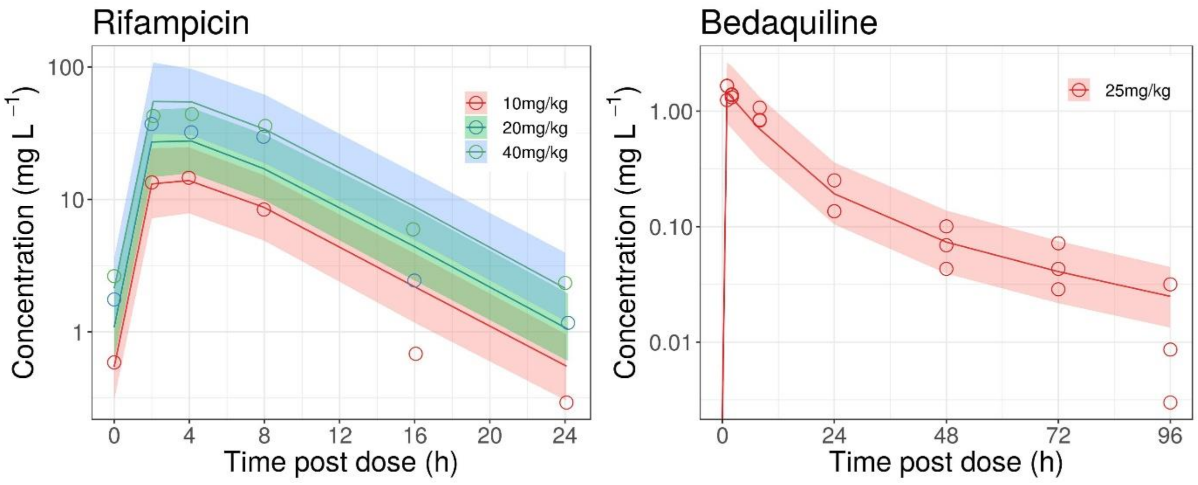

Figure S8. Visual predictive checks (VPC) of the PK models for rifampicin and bedaquiline in mice. Serum PK profiles were derived following single dose administration of bedaquiline or after 10th-13th dose of a 2-week treatment with rifampicin (given 5 days per week). Open circles depict the mean observed concentrations (3-5 mice per time point). Solid lines and shaded area represent the predicted median and 90% prediction intervals.

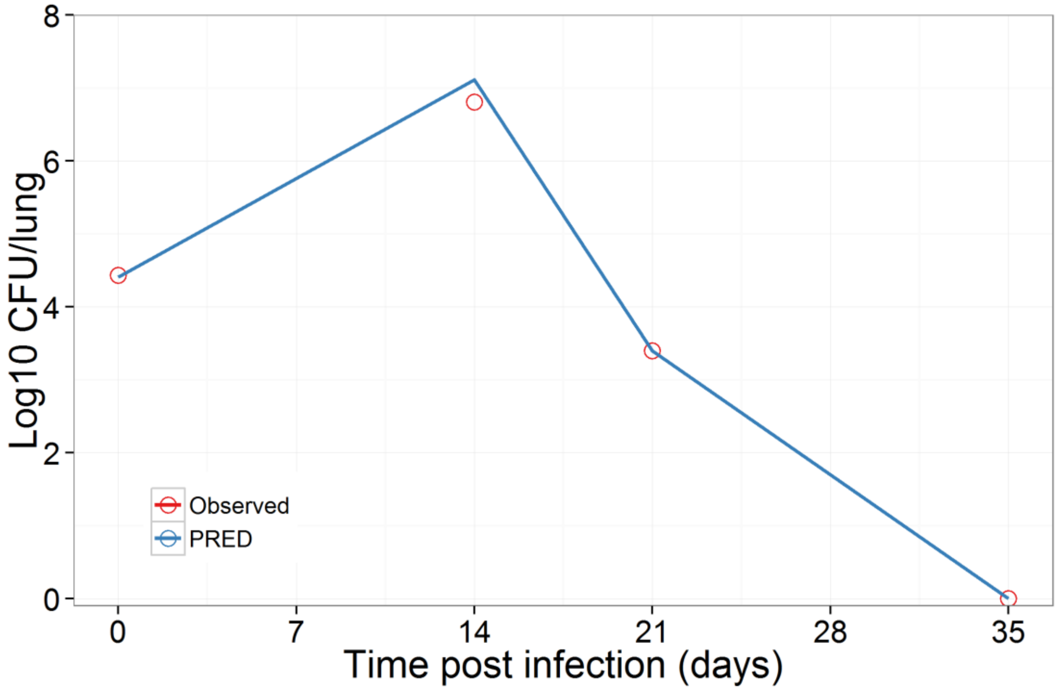

Figure S9. Model fit of the hypothetical data assumed to best represent the maximum killing rate of rifampicin in the experiment by Hu et al. (Hu, Liu, Ortega-Muro, Alameda-Martin, Mitchison & Coates, 2015). Similar to the experimental protocol used by Hu *et al.*, here it is assumed that BALB/c mice are infected via intravenous infection route followed by an incubation period of 2 weeks. To ensure that the simulated rifampicin exposure corresponded to maximum drug effect, the potency against both fast- and slow-growing *M. tuberculosis* (e.g. EC_{50}-F and EC_{50}-S) were empirically fixed to 0.001 mg/L during this estimation step, in conjunction with an artificially high dose treatment (9999 mg/kg) for 3 weeks (5 days/week). CFU = colony forming unit; PRED = population prediction.

Table S3. Overview of the estimated disease-specific parameters for each of the experiments used in the external validation.

**Parameter**	**Estimate**	**Study reference**
Inoculum (log10 CFU/lung)		
Almeida *et al.* 2009	3.33	(Almeida et al., 2009)
Hu *et al.* 2016	4.38	(Hu et al., 2016)
Rosenthal *et al.* 2012	1.72	(Rosenthal et al., 2012)
Tasneen *et al.* 2008	3.23	(Tasneen, Tyagi, Williams, Grosset & Nuermberger, 2008)
Tasneen *et al.* 2015	2.44	(Tasneen et al., 2015)
Maximum bacterial load at stationary phase, BMAX (log10 CFU/lung)		
Almeida *et al.* 2009	7.82	(Almeida et al., 2009)
Hu *et al.* 2016	7.06	(Hu et al., 2016)
Rosenthal *et al.* 2012	6.17	(Rosenthal et al., 2012)
Tasneen *et al.* 2008	7.79	(Tasneen, Tyagi, Williams, Grosset & Nuermberger, 2008)
Tasneen *et al.* 2015	6.55	(Tasneen et al., 2015)

References

Aljayyoussi G, Jenkins VA, Sharma R, Ardrey A, Donnellan S, Ward SA*, et al.* (2017). Pharmacokinetic-Pharmacodynamic modelling of intracellular Mycobacterium tuberculosis growth and kill rates is predictive of clinical treatment duration. Sci Rep 7**:** 502.

Almeida D, Nuermberger E, Tasneen R, Rosenthal I, Tyagi S, Williams K*, et al.* (2009). Paradoxical effect of isoniazid on the activity of rifampin-pyrazinamide combination in a mouse model of tuberculosis. Antimicrob Agents Chemother 53**:** 4178-4184.

Hu Y, Liu A, Ortega-Muro F, Alameda-Martin L, Mitchison D, & Coates A (2015). High-dose rifampicin kills persisters, shortens treatment duration, and reduces relapse rate in vitro and in vivo. Front Microbiol 6**:** 641.

Hu Y, Pertinez H, Ortega-Muro F, Alameda-Martin L, Liu Y, Schipani A*, et al.* (2016). Investigation of Elimination Rate, Persistent Subpopulation Removal, and Relapse Rates of Mycobacterium tuberculosis by Using Combinations of First-Line Drugs in a Modified Cornell Mouse Model. Antimicrob Agents Chemother 60**:** 4778-4785.

Raffetseder J, Pienaar E, Blomgran R, Eklund D, Patcha Brodin V, Andersson H*, et al.* (2014). Replication rates of Mycobacterium tuberculosis in human macrophages do not correlate with mycobacterial antibiotic susceptibility. PLoS One 9**:** e112426.

Rosenthal IM, Tasneen R, Peloquin CA, Zhang M, Almeida D, Mdluli KE*, et al.* (2012). Dose-ranging comparison of rifampin and rifapentine in two pathologically distinct murine models of tuberculosis. Antimicrob Agents Chemother 56**:** 4331-4340.

Swanson RV, Ammerman NC, Ngcobo B, Adamson J, Moodley C, Dorasamy A*, et al.* (2016). Clofazimine contributes to sustained antimicrobial activity after treatment cessation in a mouse model of tuberculosis chemotherapy. Antimicrob Agents Chemother 60**:** 2864-2869.

Tasneen R, Betoudji F, Tyagi S, Li SY, Williams K, Converse PJ*, et al.* (2015). Contribution of oxazolidinones to the efficacy of novel regimens containing bedaquiline and pretomanid in a mouse model of tuberculosis. Antimicrob Agents Chemother 60**:** 270-277.

Tasneen R, Tyagi S, Williams K, Grosset J, & Nuermberger E (2008). Enhanced bactericidal activity of rifampin and/or pyrazinamide when combined with PA-824 in a murine model of tuberculosis. Antimicrob Agents Chemother 52**:** 3664-3668.

Zhang T, Li SY, & Nuermberger EL (2012). Autoluminescent Mycobacterium tuberculosis for rapid, real-time, non-invasive assessment of drug and vaccine efficacy. PLoS One 7**:** e29774.
